# What Population Reveals about Individual Cell Identity: Single-Cell Parameter Estimation of Models of Gene Expression in Yeast

**DOI:** 10.1371/journal.pcbi.1004706

**Published:** 2016-02-09

**Authors:** Artémis Llamosi, Andres M. Gonzalez-Vargas, Cristian Versari, Eugenio Cinquemani, Giancarlo Ferrari-Trecate, Pascal Hersen, Gregory Batt

**Affiliations:** 1 Inria Saclay - Ile-de-France, Palaiseau, France; 2 Laboratoire Matière et Systèmes Complexes, UMR 7057, Université Paris Diderot & CNRS, Paris, France; 3 Dipartimento di Ingegneria Industriale e dell’Informazione, Università degli Studi di Pavia, Pavia, Italy; 4 Laboratoire d'Informatique Fondamentale de Lille, UMR 8022, Université de Lille 1 & CNRS, Villeneuve d'Ascq Cedex, France; 5 INRIA Grenoble - Rhône-Alpes, Montbonnot, France; ETH Zurich, SWITZERLAND

## Abstract

Significant cell-to-cell heterogeneity is ubiquitously observed in isogenic cell populations. Consequently, parameters of models of intracellular processes, usually fitted to population-averaged data, should rather be fitted to individual cells to obtain a population of models of similar but non-identical individuals. Here, we propose a quantitative modeling framework that attributes specific parameter values to single cells for a standard model of gene expression. We combine high quality single-cell measurements of the response of yeast cells to repeated hyperosmotic shocks and state-of-the-art statistical inference approaches for mixed-effects models to infer multidimensional parameter distributions describing the population, and then derive specific parameters for individual cells. The analysis of single-cell parameters shows that single-cell identity (*e*.*g*. gene expression dynamics, cell size, growth rate, mother-daughter relationships) is, at least partially, captured by the parameter values of gene expression models (*e*.*g*. rates of transcription, translation and degradation). Our approach shows how to use the rich information contained into longitudinal single-cell data to infer parameters that can faithfully represent single-cell identity.

## Author Summary

Because of non-genetic variability, cells in an isogenic population respond differently to a same stimulation. Therefore, the mean behavior of a cell population does not generally correspond to the behavior of the mean cell, and more generally, neglecting cell-to-cell differences biases our quantitative representation and understanding of the functioning of cellular systems. Here we introduce a statistical inference approach allowing for the calibration of (a population of) single cell models, differing by their parameter values. It enables to view time-lapse microscopy data as many experiments performed on one cell rather than one experiment performed on many cells. By harnessing existing cell-to-cell differences, one can then learn how environmental cues affect (non-observed) intracellular processes. Our approach is generic and enables to exploit in unprecedented manner the high informative content of single-cell longitudinal data.

## Introduction

It is well-recognized that cellular heterogeneities exist in a population of isogenic cells [1–3]. Indeed, cellular processes are noisy and generate cell-to-cell differences. Microfluidics and time-lapse fluorescence microscopy combined with cell-tracking algorithms make it possible to follow the behavior of populations of cells at the single-cell level over long time and to apply stimulations homogeneously [4,5]. Therefore, cell-cell variability in the expression of a gene of interest can be observed over extended time scales. The origins of the variability of biological processes and phenotypes are multifarious. Indeed, the observed heterogeneity of cell responses to a common stimulus is believed to originate partly from differences in cell phenotypes (age, cell size, ribosome and transcription factor concentrations, etc…), from spatio-temporal variations of the cell environments and from the intrinsic randomness of biochemical reactions. A proper assessment and modeling of such heterogeneity is therefore a challenging task since not only it has several sources but also those sources are inter-dependent and act with different strengths and on different time-scales [6].

Regarding dynamical models of gene expression, the most widely-accepted approach to take into account cell-cell variability so far relies on modeling transcription as a stochastic process [7]. Yet, these approaches only give a partial representation of cellular heterogeneity as they assume that all the measured variability originates only from the noisy expression of the modeled genes. The level of expression of other genes and their products, along with the cell’s phenotype that emerges from it, are considered as fixed in time and equal for all cells. That is, the standard modeling approach considers all gene expression noise to be intrinsic. Yet, it is known from seminal works on noise in gene expression that the overall noise breaks down into intrinsic and extrinsic components [8,9]. Although both are always present, intrinsic noise contribution is generally dominant only on short time scales and for unstable or weakly expressed proteins.

Therefore, a purely stochastic representation of cellular heterogeneity is not appropriate for a large proportion of genes and biological processes. Witnessing that validating a model encompassing both types of variability against data is still very difficult given current experimental possibilities [10], we propose to explore a different approach in which variability is represented only as stable differences among cells. This simplifying assumption is a necessary first step towards a congruent representation of the total variability in gene expression, and can be readily applied to other biological processes in which extrinsic variability dominates or when the focus lies on cellular identity.

**Fig 1:**
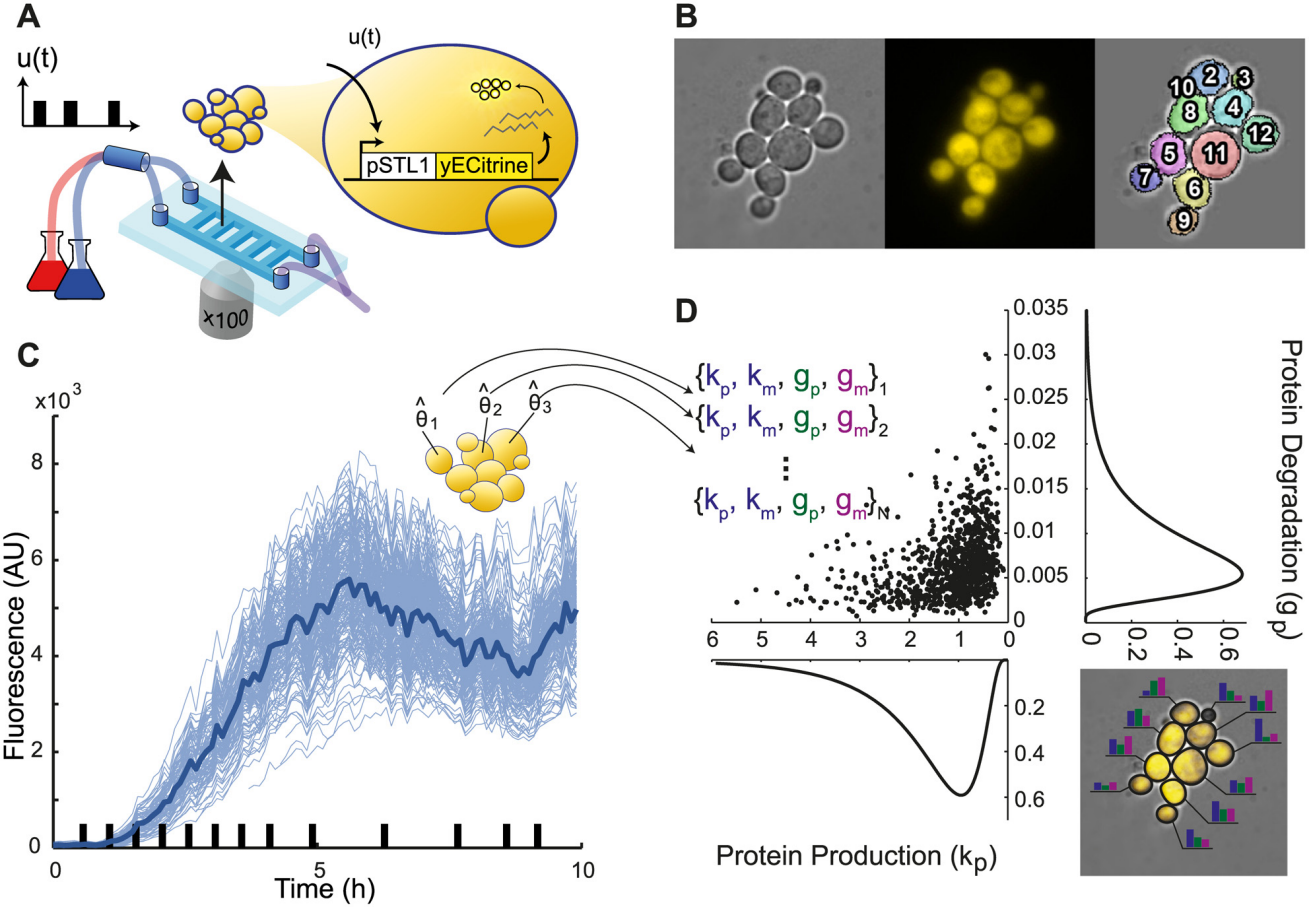
Experimental setup and principle of single-cell parameter estimation. **A**. Microfluidic device enabling the growth and imaging of yeast cells over extended durations while applying repeated hyperosmotic shocks by rapidly switching their environment between normal and hyperosmotic media. Using a reporter gene that drives the transcription of the yellow fluorescent protein yECitrine under control of the osmoresponsive promoter pSTL1, one can track the transcriptional response of cells to repeated osmotic shocks. **B**. Thanks to segmentation and tracking algorithms, the response of single-cells can be measured over several generations. **C**. As a result we obtain single-cell trajectories (thin blue lines) that show the variability in cells response to hyperosmotic stress. The thick blue line represents the median behavior. Black bars on the x-axis represent the hyperosmotic shocks applied to cells. **D**. From these trajectories, our goal is to extract the parameters of a standard model of gene expression (see text) for each cell, and therefore a multidimensional distribution describing the cell-to-cell variability. As an illustration, the right inset shows that different cells will be modeled with different parameter values to account for their own specific behavior.

Here we analyzed the temporal evolution of the level of expression of an inducible fluorescent reporter in a population of yeast cells growing in a microfluidic device. By selecting a strong inducible promoter and using a stable reporter, we placed ourselves in experimental conditions where extrinsic variability is dominant over the neglected intrinsic component. In addition we assess directly how the inferred individuality in gene expression can be related to measurable features of cell’s phenotype and physiology and therefore related to typical biological measures of cellular identity. We use a modeling approach in which, for a standard model of gene expression in yeast, each single cell is given specific parameter values while the cell population is described by a multidimensional parameter distribution (Fig 1). This leads to a challenging inference task compared to a classic situation where all cells are described by the same “mean-cell” model and parameters. Indeed the problem is shifted from obtaining a single value per parameter to obtaining parameter values for each observed cell, as well as a multidimensional distribution representing parameter values in large cell populations. This problem not only involves determining the distribution within a population for each parameter but also their mutual relationships, or more formally, their joint distribution. In order to do so, we used state-of-the-art statistical methods [11,12] that allow inferring parameters distribution across the population that are congruent with parameters attributed to each single cell. We motivate the use of such demanding statistical tools by showing why a simpler and more straightforward method is inappropriate for our current objective of representing populations by a distribution of parameters.

We propose several validations of the inference results and we analyze the obtained parameter distributions representing cell populations. Then we focus on single cells and analyze the correlation across parameters or between parameters and other single-cell features related to phenotypic and physiological variability. At last, the inheritability of the parameters of gene expression is assessed. Taken together, our results demonstrate that using the proposed framework, biologically-relevant model parameters can be attributed to individual cells and related to single-cell features, while the population of cells is represented in a concise manner. As such, this work is an important step towards identifying the major determinants of extrinsic cell-cell variability, as well as introducing quantitatively the concept of single-cell identity.

## Results

### Gene expression in response to repeated osmotic stress shows a high level of variability between cells

Using microfluidics and time-lapse microscopy we acquired longitudinal data of the response of individual yeast cells subjected to repeated hyperosmotic shocks (see Material and Methods) [13,14]. Cells were bearing a stable fluorescent reporter driven by the STL1 promoter which is strongly activated by hyperosmotic stress [15,16]. We extracted fluorescence values for large numbers of single yeast cells (typically 300) over a long period of time (typically 8–10 hours). Markedly-different behaviors were observed between individual cells (Figs 1 and S1). As extrinsic variability is arguably the dominant component of phenotypic heterogeneity in gene expression in eukaryotic cells [17,18], these differences are expected to depend at least in part on variations in the rates of transcription, translation and degradation/dilution from one cell to another. Parameters of a model of our reporter gene expression should therefore be different from one cell to another to account for extrinsic variability. By using short but pronounced and repeated inductions of gene expression with a stable reporter protein, we limited both the impact of intrinsic noise in our experiments and the deleterious effects of hyperosmotic shocks (see Experimental Design in S1 Text).

### Mixed-effects model is an ideal framework for representing extrinsic variability

Mixed-effects (ME) models are a class of statistical models introduced to describe the response of different individuals within a population to known stimuli. Here, we used a ME model where the response of individual cells was described in terms of a simple dynamical model of gene expression. Denoting with *m* and *p* the cellular level of mRNA and fluorescent protein, respectively, we have

$$\left\{ \begin{array}{l} \dot{m}(t)=k_{m}u(t)-g_{m}m(t)\\ \dot{p}(t)=k_{p}m(t)-g_{p}p(t) \end{array}\right.$$

where *u*(*t*) represents the activity of transcription factors—in our case, the phosphorylation and nuclear import of the kinase Hog1p –and is a function of the osmolarity of the cell environment (see Material and Methods and S1 Text). The production and decay rates are denoted *k*_*m*_ and *g*_*m*_ for the mRNA, and *k*_*p*_ and *g*_*p*_ for the protein, respectively. To relate fluorescence measurements to actual protein concentrations, we accounted for protein folding time using a delay *τ*. We also assumed the presence of multiplicative and additive white Gaussian measurement noise whose strength is the same for all cells (see S1 Text and S1 Table for details). Importantly, in the ME framework, it is considered that *k*_*m*_, *g*_*m*_, *k*_*p*_, and *g*_*p*_ vary within the population. Differences in parameter values may typically originate from differences in the level of key components of the gene expression machinery (*e*.*g*. RNA polymerase and ribosomes) or in environmental or physiological parameters (*e*.*g*. cell growth rate). We assumed that these parameters were log-normally distributed across the population: *θ* = (*k*_*m*_, *g*_*m*_, *k*_*p*_, *g*_*p*_) with ln(θ)~N(μ,Σ), where *μ* and Σ correspond to a vector of means and a covariance matrix, respectively. This assumption ensures the population is represented in a much more concise and general manner than what would be possible by only representing a population by the dynamics of every cell observed in an experiment.

Here, we are looking for a multidimensional distribution defined by its center of mass (*i*.*e*. a vector of mean values) and its spread (*i*.*e*., a covariance matrix) across the population. A simple, intuitive manner to tackle this problem is to search for the different parameter values that best describe each individual cell, and then compute the statistics (mean and covariance) of the underlying distribution from the set of parameter estimates. We refer to this method as the ‘*naive approach*’ since it is the natural starting point, bearing limitations that are not apparent until a proper analysis is performed. The proposed alternative is to use state-of-the-art approaches for the identification of ME models, such as Stochastic Approximation Expectation Maximization (SAEM). SAEM is a stochastic approximation version of the well-known Expectation–Maximization algorithm and has been developed for the inference of population models in presence of limited available information [11,19]. Notably SAEM is the reference approach in pharmacokinetics/pharmacodynamics studies [12,20]. However, it has not yet been applied to time-lapse single-cell data. The SAEM algorithm directly searches for multivariate distributions by alternating (i) an estimation of (an approximation of) the likelihood of the population parameters and individual observations given the current best estimate of the parameter distribution in the population and (ii) an update of the current estimate of the parameter distribution. In a second step, *a posteriori* estimates of the individual cell parameters are obtained from the inferred parameter distribution and individual data (maximum *a posteriori* estimate, MAP). This way, the fact that all parameters share (hidden) traits of the common population is explicitly taken into account. The naive and SAEM approaches are graphically represented in S2 Fig.

### The SAEM approach provides relevant and robust single-cell parameter distributions

**Fig 2:**
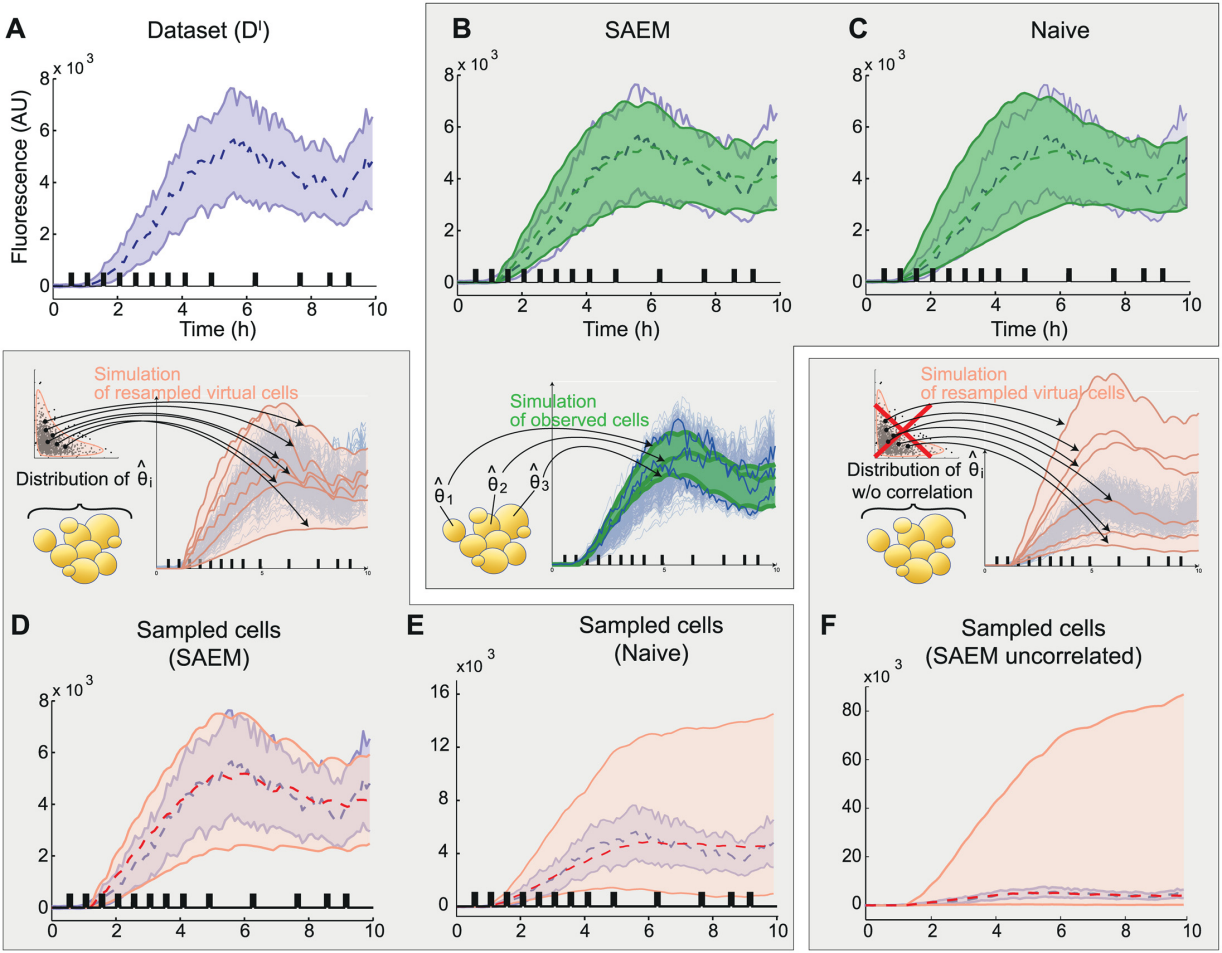
The SAEM approach provides parameter distributions that capture the population behavior because of cross-correlations between parameters. A. Representation of the experimental dataset. B. Simulated behavior obtained when using the parameters of each observed cell in the dataset (325 cells) inferred with the SAEM approach. C. Simulated behavior obtained when using the parameters of each observed cell in the dataset (325 cells) inferred with the naive approach. D. Simulated behavior of 10000 cells when resampling the population joint distribution inferred with SAEM, (pink). E. Simulated behavior of 10000 cells when resampling the population joint distribution inferred with the naive approach. F. As an illustration we show the simulated behavior of 10000 cells when resampling the population parameter distribution as in D but without preserving the covariance between parameters (*i*.*e*., using marginal distributions). For E and F, note that the y-axis has been scaled differently. Shaded areas represent the fluorescence values of 95% of the population and the dashed lines represent the median. Experimental data is represented in blue. Black bars indicate the presence of osmotic shocks. Note that unlike actual cells, all simulated cells are represented during the whole experiment (ie from 0 to 10hrs).

Both the *naive approach* and the SAEM estimation method were applied to an experimental data set comprising more than 300 cells observed during several hours. Despite the significant diversity in the behavior of individual cells (Fig 2A), both the *naive approach* and the SAEM estimation method were able to find single-cell parameters that fitted well the set of observed single-cell behaviors (Fig 2B and 2C). For the naïve approach, one can observe that the envelope of the fitted trajectories is slightly larger than the data at the early time points (Fig 2C). This simply results from the absence of data to constrain the fits at the early times for cells born during the experiment. Indeed, the average relative absolute difference between single-cell predictions and data are nearly identical in the two approaches (naïve approach: 8.7%; SAEM approach: 8.3%).

We then evaluated the capability of the obtained parameter *distributions* to actually describe the behavior of the cell population (mean and spread). To do so, the parameter distributions obtained using the *naive* and the SAEM approaches were randomly sampled, thus creating two different virtual ‘*cell populations’*, and the two corresponding sets of behaviors were computed from our model of gene expression. The SAEM-inferred parameter distribution accurately reproduced the observed behavior of the real cell population (Fig 2D), whereas the *naive approach* failed to do so (Fig 2E). Therefore, although both approaches were able to identify a set of single-cell parameters that reproduce well the behaviors of the set of observed cells, only SAEM was able to infer a parameter distribution at the population level consistent with the observed heterogeneity in gene expression.

To investigate the causes of the marked differences between the predictive power of the ME models inferred using either the naive approach or the SAEM algorithm, we compared the corresponding parameter distributions. In both cases, the mean values of the parameters were comparable and within the expected ranges (see S1 Table for parameter values and S1 Text for literature values). However, the distribution obtained with the SAEM algorithm was significantly more compact (*i*.*e*. it had a smaller volume in the parameter space) and was more structured (*i*.*e*. it had higher cross-correlations on average; S3 Fig). This strongly suggested that capturing the structure of the parameter distribution is essential in order to explain the population behavior. Both the individual statistics of each parameter, and their covariance, describing mutual relationships, contain essential information to properly account for the cell-cell variability observed in the dataset. And indeed, when using a parameter distribution with the same individual parameter statistics (mean and variance) as the distribution inferred using SAEM but with null cross-correlations (*i*.*e*. using the marginal distributions), the model lost its capability to predict the behavior of the population (compare Fig 2D and 2F). Our understanding is that in the *naive approach*, all cells are fitted individually and are subsequently *casted* into a multidimensional distribution. In contrast, SAEM allows finding equally good single-cell parameters while favoring a concise multidimensional representation of the overall population. The difference in performance between these two approaches is rooted in the fact that even with a simple model of gene expression the information contained in a single trajectory is too small to constrain the inferred parameter values in a satisfactory way. Using SAEM, we actually allow each single-cell fit to use information about the overall population, which ensures coherence between the representation of the population by distributions and of the single cells by specific parameter values. Having demonstrated that the SAEM-based identification approach captures the behavior of the cell population, from here on we focus only on the results obtained using this method.

**Fig 3:**
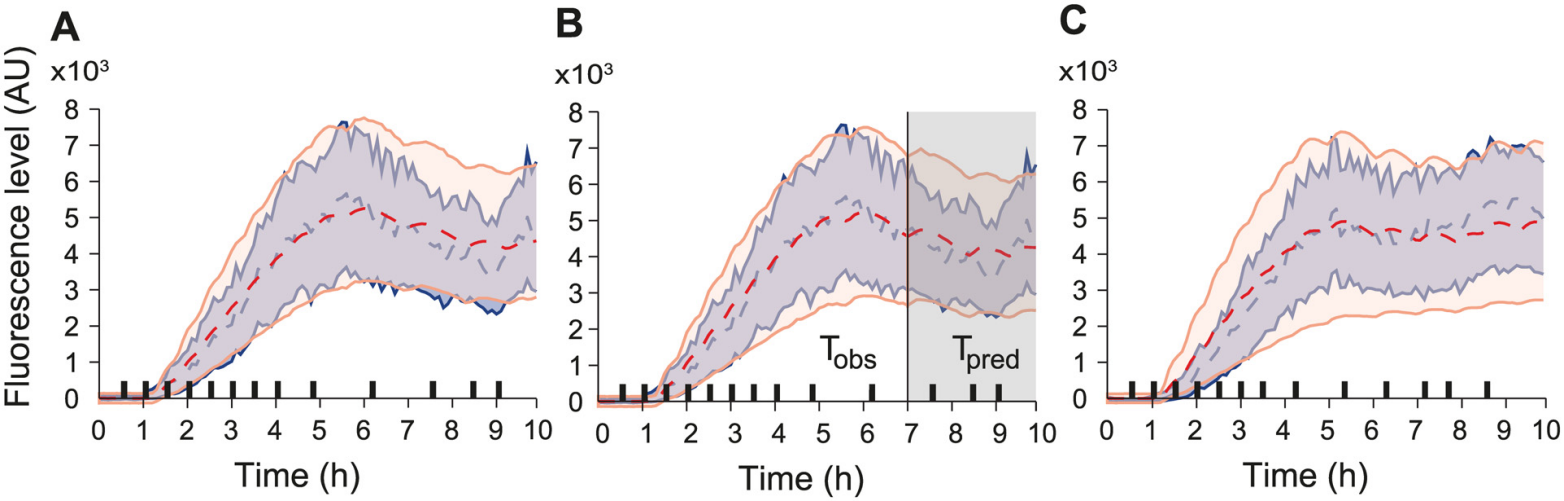
Robustness of the SAEM approach and validation of model predictive power. A Predictions obtained for a ME model having parameter distributions estimated on only 32 randomly-chosen cell trajectories (see also S2 Text). B Predictions obtained for an ME model having parameter distributions estimated using only the first 7 h of the experimental data (see also S2 Text). C Prediction obtained for the validation dataset for a ME model with parameter distributions estimated using the identification data set. Different temporal patterns of osmotic shocks were applied.

We then tested the robustness of the inference approach which is an essential property for learning algorithms. Interestingly, the performance of the SAEM inference method degraded gracefully as the number of available single-cell trajectories for identification was decreased to as few as 32 cells (Fig 3A and S2 Text), and also as the experimental time period used for learning was reduced (Fig 3B and S2 Text). Lastly, ME models with SAEM-inferred parameter distributions were still able to give good predictions when tested on a different data set (Fig 3C, see also S3 Text).

### Parameters of the gene expression model only make sense at the single-cell level

**Fig 4:**
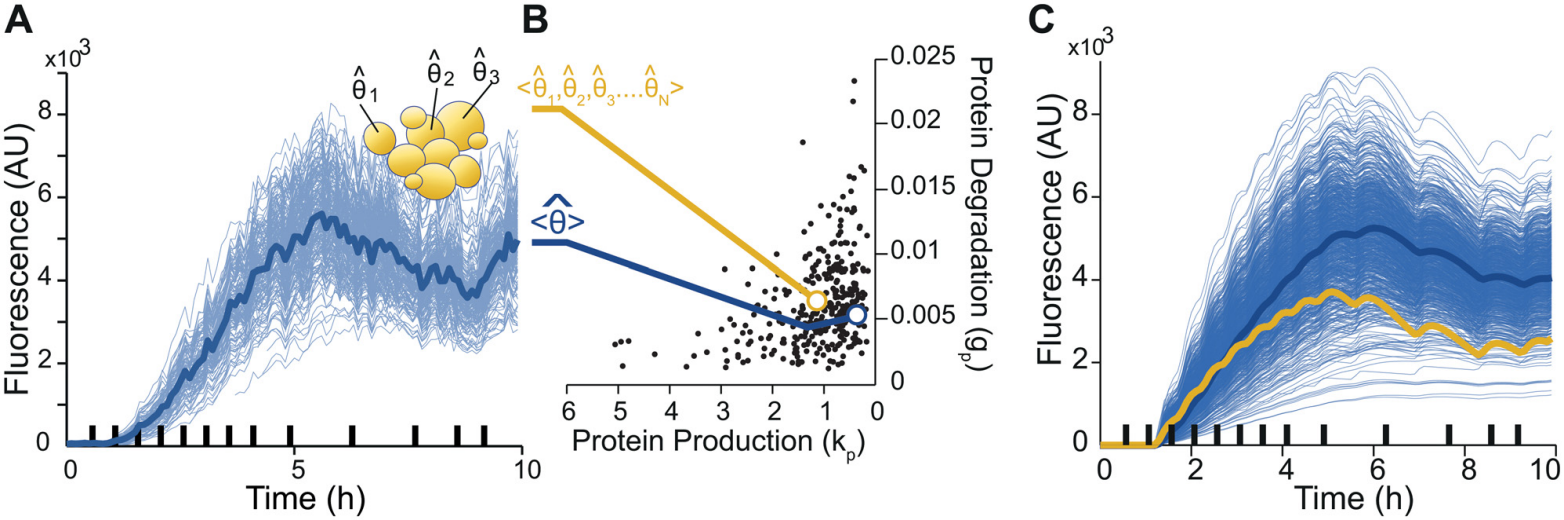
Parameters only make sense at the single-cell level. A-B. Starting from an experimental dataset (A), one can either extract the parameters that describe the average behavior (in blue), or use our framework to extract the entire collection of single-cell parameters (black dots in B) and compute the average parameters (in yellow). B-C. The average parameters do not match the parameters that best describes the average behavior. C. Visualization of 1000 simulated single-cell behaviors (blue thin lines) based on the parameters distributions shown (partially) in B. The solid blue line is a (good) simulation of the average behavior (also shown in blue in panel A). The yellow solid line is the behavior corresponding to the “average cell”, which has for parameters, the average parameters of the parameters distributions. The “average cell” behavior is clearly different from the averaged behavior.

At this point, we have showed how to efficiently and robustly extract the distributions of parameters of a standard model of gene expression from a collection of longitudinal single-cell data, and a set of parameters for each cell in the population. While we are here mostly interested in the details of the parameter distribution, we can also extract the average value for each parameter of the model. Importantly, they are different from the parameters that are obtained by fitting directly our model of gene expression to the population-averaged behavior. This is illustrated on Fig 4 where the ‘*average cell*’ trajectory (whose parameters are the average of single-cell parameters) is different from the average trajectory (obtained by directly averaging the single-cell trajectories). As mentioned in the introduction, this expected result reminds us that parameters of a model of a biological process estimated from average behaviors, as done in the vast majority of quantitative studies, may poorly represent the actual process.

### Analysis of parameter correlations may reveal non-identifiability relations

Non-identifiability arises when the information contained in data along with a model structure does not allow for the proper estimation of parameter values: several parameter values (or more usually combinations of parameter values) yield equally-good results given the available data. In our framework, very high correlations between parameter values may indicate the existence of non-identifiability relations among parameters. The first application of the SAEM algorithm showed that *k*_*m*_ and *k*_*p*_ were highly correlated, and, indeed, checking single-cell values suggested that the rates of transcription and translation could hardly, if at all, be quantified independently. A detailed identifiability analysis showed that, at the level of individual cells, these two parameters are structurally non-identifiable; only their product can be quantified (S4 Text). However, in population approaches, partial information about the second-order statistics of individual parameters can be inferred from the population statistics even if these parameters are non-identifiable at the single-cell level (S5 Text). Consequently, to address identifiability issues while preserving maximal information, we fixed the mean value for *k*_*p*_ when inferring parameter distributions using SAEM, and introduced the protein production rate *k*_*mp*_, defined as the product of *k*_*m*_ and *k*_*p*_, for the single-cell models. With these changes, shrinkage was then found to be negligible (S4 Text).

### Single-cell parameters correlate with the intensity of shocks perceived by single cells

**Fig 5:**
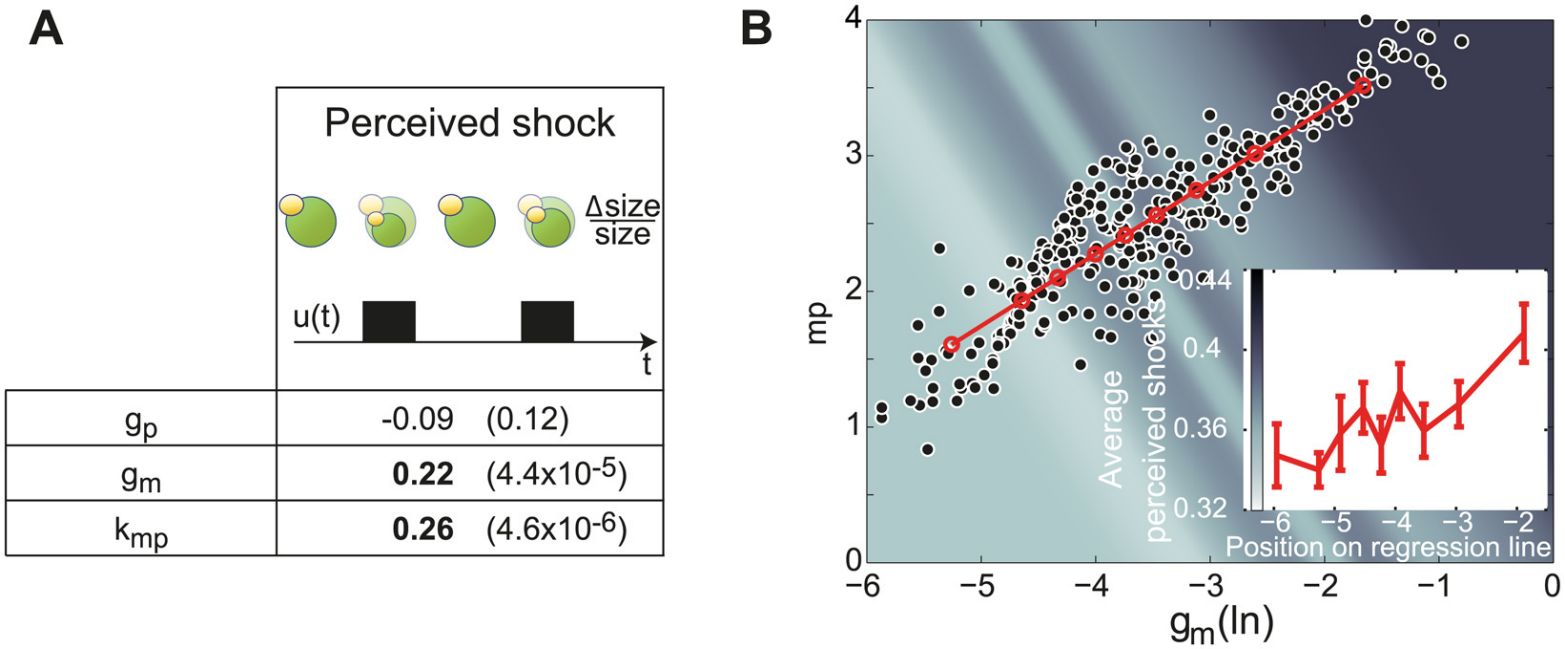
Effects of hyperosmotic shocks on intracellular processes involved in gene expression. A. Correlations between the perceived intensity of hyperosmotic shocks and single-cell parameter estimates are provided with their corresponding *p*-values (S1 Text). B. Estimated values for protein synthesis rates *k*_*mp*_ and mRNA degradation rates *g*_*m*_ for each individual cell. Their strong correlation (Spearman coefficient: 0.88; *p*-value<10^−15^) together with their mutual increase with perceived shocks intensity indicates that these two processes are jointly regulated in response to hyperosmotic shocks. Insert plot and colored background represent perceived shock intensity for 9 groups of 35 cells along the regression line.

Having identified single-cell parameter values, one may wonder whether they can be used to retrieve known facts or discover new ones on the physiology of the cell response to hyperosmotic shocks. In our model, hyperosmotic shocks affect all cells identically. However, in the microfluidic device, the intensity of the shock perceived by different cells varied, as evidenced by differences in the reduction of cellular volume following shocks. Therefore, one should find that protein production parameters inferred for the most severely impacted cells are statistically higher than average. We thus estimated the perceived shock intensities as the time-averaged reduction of cellular volume following shocks, and compared for all the cells the inferred parameter values and the perceived shock intensities. We found a strong correlation between protein production rates and shock intensities in agreement with our hypothesis. Moreover an equally-strong correlation was also found with mRNA degradation rates (Fig 5A). This second feature, obtained by our framework without any additional measurements or hypothesis, is consistent with the known global destabilization of mRNAs observed after hyperosmotic shocks [21]. Lastly, the simultaneous increase of protein production rates and mRNA degradation rates strongly correlates with the increase of the perceived shock (Fig 5B) indicating that these two processes are jointly regulated in response to hyperosmotic shocks. Note that the direct experimental identification of such co-variations would be very challenging. This shows the interest of extracting and analyzing distributions of model parameters for the identification of joint regulations.

### Single-cell parameters correlate with single-cell physiological features

**Fig 6:**
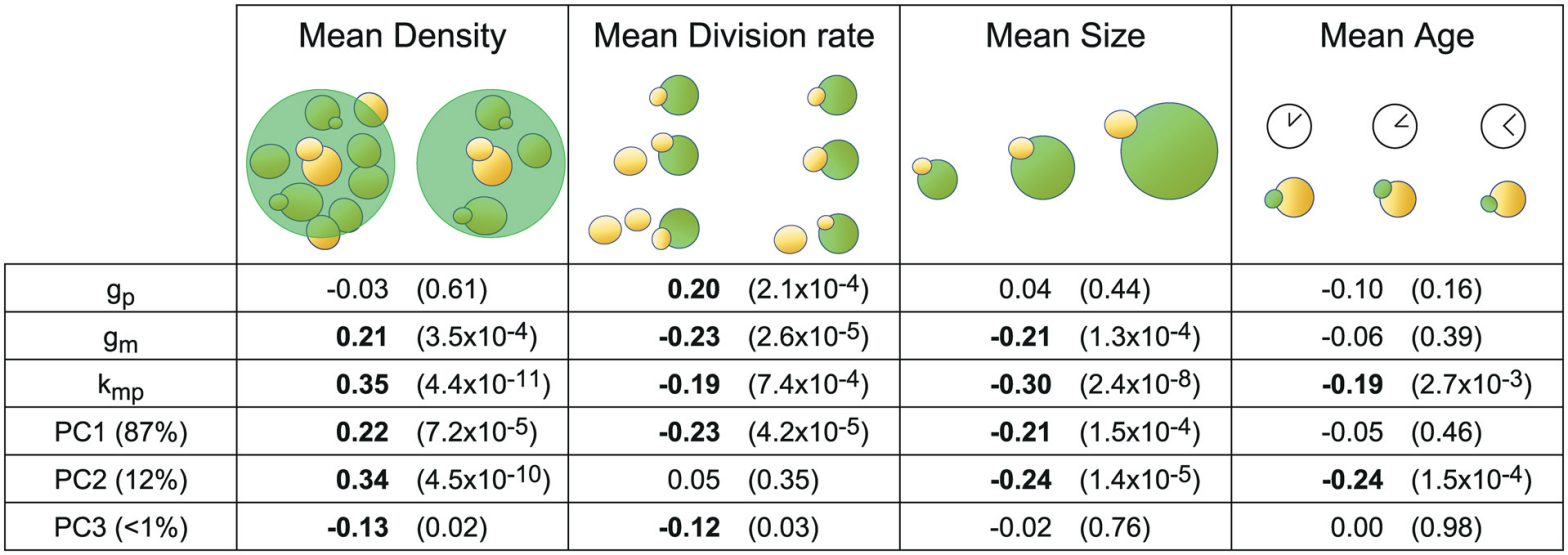
Harnessing cell-to-cell variability reveals correlations between parameter values and independently-measured cellular features. Local cellular density, division rate, size and age were quantified with single-cell resolution (S1 Text). Correlations between these single-cell features and the single-cell parameter estimates and their principal components are provided with their corresponding *p*-values. Note the expected correlation between protein degradation/dilution rate *g*_*p*_ and the cell division rate. The proportion of variance accounted for by each principal component is indicated in parenthesis.

In addition to hyperosmotic shocks, several features related to the cell physiology or local environment are also expected to relate to gene expression [22]. Such features notably include cell division rate, cell size, cell age, and local cell density. Since these features can be measured or estimated for each single-cell based on bright-field time-lapse imaging, one can again harness cell-to-cell variability and search for relations between these features and the parameters that describe intracellular processes involved in gene expression. Firstly, we searched for a correlation between the protein decay parameter, *g*_*p*_, and the cell division rate. Indeed, as the fluorescent reporter we used has a long half-life and photobleaching is negligible (see Initial parameters values S1 Text), one should expect that its observed decay comes mostly from dilution due to cellular growth. Therefore, we quantified for each cell its division rate, averaged over the observation period (S1 Text) and, as expected, found a significant positive correlation between the measured average single-cell division rate and the protein decay parameter *g*_*p*_ (Fig 6). Stated differently, using exclusively the fluorescence profile of individual cells and the inferred parameter distribution for the cell population as an *a priori*, the inference approach attributed statistically higher dilution rates to cells that grow faster. Several other highly significant correlations between single-cell parameters and the above-mentioned single-cell measured features were observed (Fig 6). Note that all measured features were averaged across time to allow the comparison with the time-invariant model parameters (S1 Text). Although it is difficult to attribute in a systematic manner a direct and unambiguous biological interpretation of the observed correlations between coarse-grained model parameters and cell features, one can nevertheless observe (i) that cell density appears to have a pronounced influence on the protein production rate, suggesting that—even in microfluidic growth chambers—the environment of the cells should not be assumed to be perfectly homogeneous, and (ii) that the correlations of the protein production rates and mRNA degradation rates with every measured feature always have the same sign, corroborating the presence of mechanisms for the joint regulation of these processes in our system.

More generally, one wonders how the different measured cell features relate to the overall (multivariate) parameter variability. We conducted a principal component analysis (PCA) of the set of inferred single-cell parameter values. This yielded a new parameterization of the model (new parameters being called principal components PC1, PC2 and PC3) that is particularly relevant to investigate variability as, unlike natural parameters, each principal component is uncorrelated to the others. The analysis showed that the first two components PC1 and PC2 represented 87% and 12%, respectively, of the overall variance in single-cell parameter values, and that these principal components correlated very significantly with measured cell features. We then ranked the various features based on their correlation with the variability captured by the inferred ME model. For a given feature, this is defined as the weighted average correlation with the different PCs, with weights equal to the importance (*i*.*e*., variance) of every PC. It appeared that local cell density was the most important factor (average correlation: 0.23), followed by cell size (0.21) and the division rate (0.2). To our knowledge, there is no established direct connection between local cell density and gene expression in yeast. It would be interesting to investigate this connection at the molecular level. Quite surprisingly, from our data, age was not associated with a significant variability in parameter values. Taken together, our results show that, for quantitative studies, features other than colony growth rate should be taken into account. A natural extension of this study would be to investigate how the inclusion of these features in the model, seen as covariates, could improve single-cell predictions.

### Single-cell parameters are partly inherited from mother to daughter

**Fig 7:**
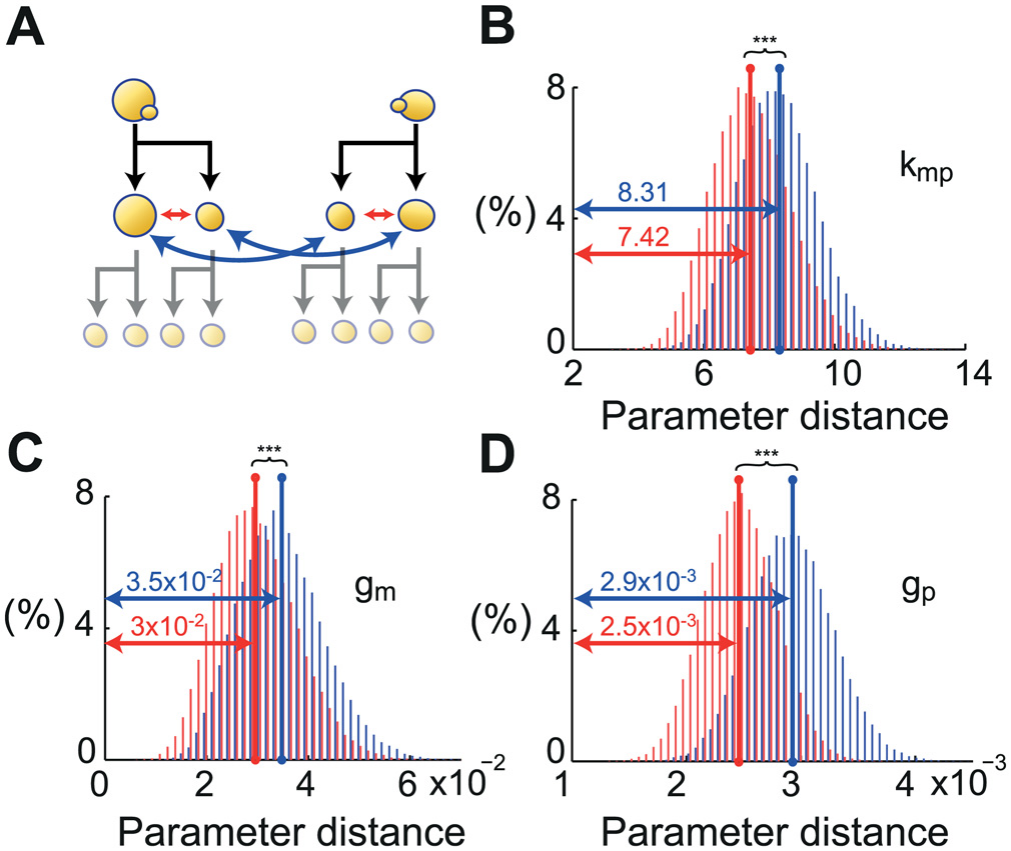
Parameter values of individual cells are statistically closer to the parameters of their own mother than to the parameters of another mother cell. (A) The distance between parameters of related mother and daughter cells (MD) and non-related mother and daughter cells (nMD) were compared. (B-D) Distribution for each parameter of the average distance between 40 pairs of MD (red) and nMD (blue) for 50000 combinations obtained by bootstrapping (S1 Text). All parameters are closer between mothers and daughters than on average (*** *p*-values < 10^−15^).

Finally, we investigated inheritance of single-cell parameters. Statistical tests showed that the parameters of mother and daughter cells were significantly closer to each other than the parameters of random cell pairs (S1 Text and S4 Fig). However, this comparison does not exclusively test the effect of lineage. The fact that mother and daughter cells share a similar environment may also explain this result. To study the specific influence of lineage, we compared the parameter values between pairs of cells that either were mother and daughter (related mother/daughter pairs) or were a mother and the unrelated daughter of another mother cell (non-related mother/daughter pairs), with all cells growing in the same microfluidic chamber so as to limit environmental bias. As shown in Fig 7, the parameter values of individual cells were statistically closer to the parameters of their own mother cell than to the parameters of another mother cell. It appears that parameter values are 16% (resp. 14%, 10%) closer in genuine mother/daughter pairs for *g*_*p*_ (resp. *g*_*m*_, *k*_*mp*_). Although mild in absolute terms, bootstrapping testing showed the presence of a statistically strong inheritance effect (*p*-values < 10^−15^ for all parameters, S1 Text). Importantly, we verified using a more restrictive notion of nMD pairs that the observed inheritance effect was not due to the fact that mother and daughter cells have more similar mean densities on average than nMD cells since the former share the same environment. Interestingly, we also found that daughter cells are on average 14% more sensitive than their mothers and that the intensity of the perceived shocks is anti-inherited: the most resistant mothers have the most sensitive daughters, and conversely.

## Discussion

In this work, we proposed an approach for capturing the biological variability observed in single-cell time-lapse microscopy experiments by *distributions* of parameters. By doing so, we address a fundamental issue encountered in the vast majority of quantitative studies where parameters of deterministic or stochastic models of intracellular processes make sense at the single-cell level but are estimated for a virtual ‘mean cell’. The analysis was based on the mixed-effects (ME) modeling framework and two inference approaches were evaluated. The relevance of the ME framework for modeling biological processes has been recently recognized [23,24]. The use of advanced statistical methods, like SAEM, was essential to properly capture the variability of the biological parameters across the population in a simple manner, including most notably the correlation among them. In addition, we showed that the SAEM method scales to real-life problems and provides robust results. With this approach, the information on each and every cell is jointly used to calibrate the model parameter distribution, alleviating the problem of limited observability and noisy observations encountered at the individual-cell level. We then demonstrated the biological relevance of the inferred cell-specific parameters, as they were partly inherited from mother to daughter cells and correlated with independently-measured single-cell features.

Our approach is adapted to calibrate models explicitly accounting for extrinsic variability. From a mechanistic viewpoint, two components of biological variability, termed intrinsic and extrinsic noise, have been proposed. For a given cellular process, intrinsic variability is mostly related to fast fluctuations coming from stochasticity in molecular reactions while extrinsic variability includes more stable cell-to-cell differences in intracellular and extracellular environments [8,17,25]. Thanks to recent methodological developments, such as finite state truncation methods, significant progress have been made in the identification of intrinsic noise models, in particular for the study of gene expression [26]. Such models assume that the different observations arise from different realizations of the same stochastic process and, therefore, are still based on the notion of a virtual mean—although noisy—cell. In comparison, and despite recent methodological developments [27,28], few attempts have been made to infer extrinsic noise models from data, see [4,10,23,29,30] and our previous work [31]. We refer the reader to Karlsson et al. [24] for a detailed discussion of these works. This is surprising, given the fact that extrinsic noise has been shown to be the dominating component in many biological systems [17,18,32] and that application of cell population models has proven extremely useful, notably to explain cell decision processes [3]. Moreover, with the notable exceptions of Zechner *et al* [10] and Gonzalez *et al* [31], no method that exploits single-cell time-lapse data for the identification of cell population models has been able to predict population behaviors. Interestingly, Zechner *et al* [10] proposed a very general framework capturing intrinsic and extrinsic variability by using a stochastic model based on the chemical master equation with parameter distributions. They investigated whether this modeling framework was able to capture both noise components appropriately, all of the extrinsic variability being aggregated into a unique cell-dependent parameter. Here, we pursued a different objective. We focused on extrinsic noise and investigated whether multidimensional parameter distributions provide an accurate description thereof and can be inferred from the available experimental data, whether the inferred single-cell parameter values are biologically-relevant, and how extrinsic noise is distributed across different cellular processes. Given the identifiability issues encountered already on relatively simple ME models, one might wonder whether more complex models combining the use of a stochastic interpretation of the reactions and of distributions for all (or most of) the parameters can be accurately identified based on available experimental data. Another attractive possible extension of the mixed-effect framework is to replace the purely static description of cell-to-cell differences obtained by using different, time-invariant parameter values by a more dynamical representation using reaction parameters that slowly fluctuate in time. This can typically be done by accounting for the stochastic turnover of the proteins underlying the various reactions involved in the processes of interest [33].

The possibility of identifying single-cell models opens new perspectives. Indeed, our results support the approach advocated by Pelkmans and coworkers *(18)* in which "*studying cell-to-cell variability [*…*] will increase our understanding of how cellular activities are embedded in the physiology of a cell*.*"* Following what we have shown here, one could dissect the variability of the different cellular processes involved in a particular phenotypic response and search for correlations with different cellular processes and with environmental factors. Such rich information on the integrated functioning of cells is otherwise barely accessible. More fundamentally, single-cell modeling provides a quantitative tool to study the notion of *cell identity*, as it offers a quantitative description of cell-to-cell differences. Lastly, to which extent this increased knowledge can be used to improve our ability to predict and ultimately control single-cell behavior is a question of interest for both the systems and synthetic biology communities [14,34–36].

## Materials and Methods

### Yeast strain and microscopy

All experiments were performed using a STL1::yECitrine-HIS5, Hog1-mCherry-hph yeast strain derived from the S288C background [14]. Cells were cultured overnight in synthetic complete (SC) medium at 30°C, in a shaking incubator at 250 rpm, and then the cultures were diluted in SC so as to reach an optical density of ~0.2 in 4h. Exponentially-growing cells were injected into a home-made microfluidic device [14]. Liquid medium was flowed using a peristaltic pump (IPC-N, Ismatec) placed after the microfluidic device (flow rate: 120μL/min). A computer-controlled three-way valve (LFA series; The Lee Company) was used to select between normal medium (SC) or the same medium supplemented with 1M sorbitol. The microfluidic chip was made by casting polydimethylsiloxane (PDMS; Sylgard 184 kit; Dow Corning) on a master wafer (made by soft lithography), curing it at 65°C overnight, pealing it off, and bonding it to a glass coverslip after plasma activation. The device has 5 chambers of 200x400x3.6 μm where cells are imaged. These chambers are connected to larger channels where medium flows such that the environment of the imaging chamber is changed by diffusion only (see [14]). After having loaded cells in the device, we leave them to rest with SC flowing for 30 min before starting the experiment. A switch of the valve state did not lead to an instantaneous change of the cells’ environment inside the microfluidic device: ~2 min were needed for the fluid to pass from the valve to the channels and the imaging chamber.

The cells were imaged using an automated inverted microscope (IX81; Olympus) equipped with an X-Cite 120PC fluorescent illumination system (EXFO) and a QuantEM 512 SC camera (Roper Scientific). The temperature of the microscope chamber, which also contains the media reservoirs, was constantly held at 30°C by a temperature control system (Life Imaging Services). All of these components were driven by the open-source software μManager which was interfaced with Matlab. Images were taken using a 100× oil immersion objective (PlanApo 1.4 NA; Olympus). The fluorescence exposure time was 200 ms, with fluorescence illumination intensity set to 50% of maximal power. The fluorescence exposure time was chosen such that the fluorescent illumination did not cause noticeable effects on cellular growth over extended periods of time. Importantly, illumination, exposure time, and camera gain were not changed between experiments, and besides background and auto-fluorescence subtraction (defined as the minimum intensity in the first frame), no data renormalization or processing was done. Imaging was performed at a frequency of one frame every 3 min for bright-field and one frame every 6 min for fluorescence measurements. The duration of the experiments was 10 hours.

### Measurements of gene expression and physiological features at the single-cell level

Single-cell gene expression profiles were obtained in two experiments: one for identification ($D^{I}$; 325 single-cell trajectories) and one for validation ($D^{V}$; 166 single-cell trajectories). The randomly-generated profiles of hyperosmotic stresses differed in each experiment. Image analysis was performed using a home-made segmentation and tracking tool, CellStar. After observing that newly-detected cells usually corresponded to buds still attached to their mother for a long period of time after detection and might present fluorescence quantification artifacts (due to their small size and variable focus), we discarded the information obtained during the first two hours for new cells. Only cells imaged for more than 5 h were selected for identification and validation. The average size of a cell corresponds to its size measured at each time point in bright-field images and averaged over all time points. Average cell age and density were defined analogously. The density of the environment of a single cell was defined as the area occupied by neighbor cells relative to the area of the neighborhood of the cell. The neighborhood was defined as a disk with a radius corresponding to five times the radius of a typical cell. The relative changes in the size of the cells caused by budding events were used to estimate single-cell division times from bright-field images and compute the average cell specific division rate. After automated segmentation and tracking, lineage was manually extracted from the microscopy images. More details are provided in S1 Text.

### Single-cell models and ME population models

We assumed that the transcription factor activity, *u*(*t*), depends on the osmolarity effectively sensed by the cells inside the microfluidic chambers, *u*_*c*_(*t*), which itself depends on the valve status, *u*_*v*_(*t*) (S1 Text). To relate fluorescence measurements to actual protein concentrations, we accounted for protein maturation time using a delay *τ* and assumed the presence of multiplicative and additive measurement noises that are white and Gaussian (S1 Text). A mixed-effects population model is then obtained from single-cell models by assuming that the parameters of the population of cells follow log-normal distributions. More details on the modeling assumptions are provided in S1 Text.

### Inference of single-cell and ME population models

Two methods were proposed to infer ME population models: a naive approach and SAEM. The naive approach used the local optimization algorithm fminsearch from Matlab to maximize the (log-)likelihood of the parameters tested, given the observed data for the considered cell. The parameter distribution for the ME model is then defined based on the set of single-cell parameters. The SAEM approach aims directly at maximizing the likelihood of the population (high-level) parameters describing the distributions of the model parameters, given all the single-cell data. We used the SAEM implementation of Monolix software. Lastly, having inferred a distribution for the model parameters of a population of cells, one could estimate the most likely parameter values for each single cell (ME single-cell models). We used the local optimization tool fminsearch from Matlab to implement a maximum *a posteriori* approach. For more details on the parameter inference approach see S1 Text.

### Relating the specific intracellular processes involved in gene expression with other, non-modeled cellular properties

The analysis of the correlations between the perceived shocks or the single-cell measured features and the estimated parameters was performed using the Spearman coefficient of correlation. The significance of the correlations (*p*-values) was assessed using the standard two-tailed test implemented in the Matlab statistics toolbox. To test whether parameters of mother and daughter cells were statistically closer than on average, we constructed pairs of cells that differed solely by whether they were direct relatives (mother/daughter pairs, MD pairs) or not (non-related mother/daughter pairs, nMD pairs). The comparison of the mean distance between MD pairs and nMD pairs was performed by bootstrapping (S1 Text).

## Supporting Information

S1 Text Supplementary Materials and Methods. Additional information on experimental design, data analysis, estimation of single cell quantitative features, cell lineage reconstruction, modeling of the osmostress-induced gene expression, parameter inference, simulation of population behavior, correlation with quantitative single-cell measurements, and heritability analysis.
(PDF)

S2 Text Robustness of population predictions. Influence of the cell number and of the learning time horizon.
(PDF)

S3 Text Validation of population predictions. Predicting population behavior on two validation data sets.
(PDF)

S4 Text Identifiability analysis. Parameters *k*_*m*_ and *k*_*p*_ cannot be assigned values unambiguously no matter the quality and quantity of fluorescence measurements.
(PDF)

S5 Text On learning the statistics of non-identifiable parameters. Statistical properties of parameters that are not distinguishable at the single-cell level can nevertheless be constrained in a population approach.
(PDF)

S1 FigpSTL1 expression in response to repeated osmotic stresses shows a high level of variability between cells.A. Minimum, maximum and average cellular fluorescence levels in the identification dataset $D^{I}$. Back bars represent input shocks. B. Set of single cell trajectories present in the identification dataset $D^{I}$ (solid lines). Samples that did not pass all quality tests described in S1 Text appear as light blue dots. C. Set of single cell trajectories present in the validation dataset $D^{V}$.(PDF)

S2 Fig Statistical inference methods for single-cell and population parameter estimation. In the naive approach, optimization is used to seek -for each cell- parameter values fitting the individual behavior of the cell via residual minimization (top, step 1). The distribution describing all of the estimated parameter values is then deduced (top, step 2). In the proposed method, the SAEM tool is used to infer a distribution that explains the set of individual behaviors at the distribution level (bottom, step 1). Parameter values for single cells are then estimated based on the particular behavior of the cell and the inferred distribution for the population, using maximum *a posteriori* estimation (bottom, step 2).
(PDF)

S3 Fig The distribution that better describes the entire population is more compact and more structured. A. 2D plot describing the distribution of the (logarithm of) single-cell parameters for two parameters (insert: same data shown in natural scale). The ellipses represent the region in which 50% of the parameters are distributed. B. Two metrics were computed to quantify the difference in the structure of the parameter distributions at a more global level. The first metric was the average of the coefficients of the variation matrix (i.e. of the off-diagonal terms cov_*ij*_/(*μ*_*i*_*μ*_*j*_) between the parameters of the model; this represents the amount of structure in the parameter distribution and shows that SAEM yielded a more structured parameter distribution. The second metric was the volume in the parameter space of the 95%-confidence ellipsoid associated with the covariance matrix. This yields a measure of the typical volume of parameter space occupied by the parameter distribution, and therefore, quantifies the spread of the parameter distributions. This showed that the SAEM approach described the population with a smaller distribution.
(PDF)

S4 FigAverage parameter distance of Mother-Daughter pairs against random pairs from the same experiment.The blue bar represent the average distance in parameters between 55 mother-daughter pairs from experiment $D^{I}$. The red distribution is obtained by bootstrapping 20000 sets of 55 random pairs of cells (from the same experiment). We see that the distance is very significantly smaller for mother-daughter pairs.(PDF)

S1 TableParameter estimates for the mixed-effects model using the naive inference approach (A), using SAEM on the identification dataset $D^{I}$ (B) and using SAEM on the validation dataset $D^{V}$ (C).(A) Initial values for the search have been obtained by global optimization (CMAES) on the mean behavior starting from literature-based parameters. The value of the delay *τ* has been fixed for all cells to its mean-cell. Therefore, statistics on its variability have been shaded. The dataset used is the identification set $D^{I}$ (B and C) The parameter search is initialized with parameter means extracted from the literature and a diagonal covariance matrix. The parameter search has been adapted to account for the structural non-identifiability relation of *k*_*m*_ and *k*_*p*_ (only their product is relevant in single-cell models): the mean of *k*_*p*_ is kept at a constant value during the search. No constraints are placed on its variance though. The value of the delay *τ* is estimated but is set identical for all cells. The dataset used for identification is $D^{I}$ (B) and $D^{V}$ (C). The relative standard errors of the estimated moments are typically less than 2%, with the exception of the estimate of *SD*[*k*_*m*_] where it was 8%.(PDF)

S1 DataData files containing raw fluorescence values for all individual cells tracked in the $D^{I}$ and $D^{V}$ experiments.(ZIP)

## References

[1] Balázsi G, van Oudenaarden A, Collins JJ. Cellular decision making and biological noise: from microbes to mammals. Cell. 2011;144: 910–25. 10.1016/j.cell.2011.01.030 21414483 PMC3068611

[2] Raj A, van Oudenaarden A. Nature, Nurture, or Chance: Stochastic Gene Expression and Its Consequences. Cell. 2008;135: 216–226. 10.1016/j.cell.2008.09.050 18957198 PMC3118044

[3] Spencer SL, Gaudet S, Albeck JG, Burke JM, Sorger PK. Non-genetic origins of cell-to-cell variability in TRAIL-induced apoptosis. Nature. 2009;459: 428–32. 10.1038/nature08012 19363473 PMC2858974

[4] Spiller DG, Wood CD, Rand DA, White MRH. Measurement of single-cell dynamics. Nature. 2010;465: 736–45. 10.1038/nature09232 20535203

[5] Locke JCW, Elowitz MB. Using movies to analyse gene circuit dynamics in single cells. Nat Rev Microbiol. 2009;7: 383–92. 10.1038/nrmicro2056 19369953 PMC2853934

[6] Huang S. Non-genetic heterogeneity of cells in development: more than just noise. Development. 2009;136: 3853–3862. 10.1242/dev.035139 19906852 PMC2778736

[7] Paulsson J. Models of stochastic gene expression. Phys Life Rev. 2005;2: 157–175.

[8] Elowitz MB, Levine AJ, Siggia ED, Swain PS. Stochastic gene expression in a single cell. Science. 2002;297: 1183–1186. 12183631 10.1126/science.1070919

[9] Raser JM, O’Shea EK. Noise in gene expression: origins, consequences, and control. Science. 2005;309: 2010–3. 10.1126/science.1105891 16179466 PMC1360161

[10] Zechner C, Unger M, Pelet S, Peter M, Koeppl H. Scalable inference of heterogeneous reaction kinetics from pooled single-cell recordings. Nat Methods. 2014;11: 197–202. 10.1038/nmeth.2794 24412977

[11] Kuhn E, Lavielle M. Maximum likelihood estimation in nonlinear mixed effects models. Comput Stat Data Anal. 2005;49: 1020–1038.

[12] Lavielle M. Mixed Effects Models for the Population Approach. CRC Press; 2014.

[13] Hersen P, McClean MN, Mahadevan L, Ramanathan S. Signal processing by the HOG MAP kinase pathway. Proc Natl Acad Sci U S A. 2008;105: 7165–70. 10.1073/pnas.0710770105 18480263 PMC2386076

[14] Uhlendorf J, Miermont A, Delaveau T, Charvin G, Fages F, Bottani S, et al Long-term model predictive control of gene expression at the population and single-cell levels. Proc Natl Acad Sci U S A. 2012;109: 14271–14276. 10.1073/pnas.1206810109 22893687 PMC3435223

[15] O’Rourke SM, Herskowitz I. Unique and redundant roles for HOG MAPK pathway components as revealed by whole-genome expression analysis. Mol Biol Cell. 2004;15: 532–542. 14595107 10.1091/mbc.E03-07-0521 PMC329229

[16] Ferreira C, van Voorst F, Martins A, Neves L, Oliveira R, Kielland-Brandt MC, et al A member of the sugar transporter family, Stl1p is the glycerol/H+ symporter in Saccharomyces cerevisiae. Mol Biol Cell. 2005;16: 2068–2076. 15703210 10.1091/mbc.E04-10-0884 PMC1073684

[17] Raser JM, O’Shea EK. Control of stochasticity in eukaryotic gene expression. Science. 2004;304: 1811–1814. 15166317 10.1126/science.1098641 PMC1410811

[18] Pedraza JM, van Oudenaarden A. Noise Propagation in Gene Networks. Science. 2005;307: 1965–1969. 15790857 10.1126/science.1109090

[19] Delyon B, Lavielle M, Moulines E. Convergence of a stochastic approximation version of the EM algorithm. Ann Stat. 1999;27: 94–128.

[20] Chan PLS, Jacqmin P, Lavielle M, McFadyen L, Weatherley B. The use of the SAEM algorithm in MONOLIX software for estimation of population pharmacokinetic-pharmacodynamic-viral dynamics parameters of maraviroc in asymptomatic HIV subjects. J Pharmacokinet Pharmacodyn. 2011;38: 41–61. 10.1007/s10928-010-9175-z 21088872 PMC3020311

[21] Romero-Santacreu L, Moreno J, Pérez-Ortín JE, Alepuz P. Specific and global regulation of mRNA stability during osmotic stress in Saccharomyces cerevisiae. RNA. 2009;15: 1110–20. 10.1261/rna.1435709 19369426 PMC2685517

[22] Snijder B, Pelkmans L. Origins of regulated cell-to-cell variability. Nat Rev Mol cell Biol. 2011;12: 119–25. 10.1038/nrm3044 21224886

[23] Almquist J, Bendrioua L, Adiels CB, Goksör M, Hohmann S, Jirstrand M. A Nonlinear Mixed Effects Approach for Modeling the Cell-To-Cell Variability of Mig1 Dynamics in Yeast. PLoS One. 2015;10: e0124050 10.1371/journal.pone.0124050 25893847 PMC4404321

[24] Karlsson M, Janzén DLI, Durrieu L, Colman-Lerner A, Kjellsson MC, Cedersund G. Nonlinear mixed-effects modelling for single cell estimation: when, why, and how to use it. BMC Syst Biol. BMC Systems Biology; 2015;9: 52 10.1186/s12918-015-0203-x 26335227 PMC4559169

[25] Hilfinger A, Paulsson J. Separating intrinsic from extrinsic fluctuations in dynamic biological systems. Proc Natl Acad Sci U S A. 2011;108: 12167–72. 10.1073/pnas.1018832108 21730172 PMC3141918

[26] Neuert G, Munsky B, Tan RZ, Teytelman L, Khammash M, van Oudenaarden A. Systematic identification of signal-activated stochastic gene regulation. Science. 2013;339: 584–7. 10.1126/science.1231456 23372015 PMC3751578

[27] Hasenauer J, Waldherr S, Doszczak M, Radde N, Scheurich P, Allgöwer F. Identification of models of heterogeneous cell populations from population snapshot data. BMC Bioinformatics. BioMed Central Ltd; 2011;12: 125 10.1186/1471-2105-12-125 PMC3114742 21527025

[28] Hasenauer J, Hasenauer C, Hucho T, Theis FJ. ODE constrained mixture modelling: a method for unraveling subpopulation structures and dynamics. PLoS Comput Biol. 2014;10: e1003686 10.1371/journal.pcbi.1003686 24992156 PMC4081021

[29] Bonassi F V, You L, West M. Bayesian learning from marginal data in bionetwork models. Stat Appl Genet Mol Biol. 2011;10: 49 10.2202/1544-6115.1684 PMC3215428 23089812

[30] Zechner C, Ruess J, Krenn P, Pelet S, Peter M, Lygeros J, et al Moment-based inference predicts bimodality in transient gene expression. Proc Natl Acad Sci U S A. 2012;109: 8340–8345. 10.1073/pnas.1200161109 22566653 PMC3361437

[31] Gonzalez AM, Uhlendorf J, Cinquemani E, Batt G, Ferrari-Trecate G. Identification of biological models from single-cell data: A comparison between mixed-effects and moment-based inference. Proc 12th IEEE Eur Control Conf. IEEE Press; 2013; 3652–3657.

[32] Colman-Lerner A, Gordon A, Serra E, Chin T, Resnekov O, Endy D, et al Regulated cell-to-cell variation in a cell-fate decision system. Nature. 2005;437: 699–706. 10.1038/nature03998 16170311

[33] Bertaux F, Stoma S, Drasdo D, Batt G. Modeling dynamics of cell-to-cell variability in TRAIL-induced apoptosis explains fractional killing and predicts reversible resistance. PLoS Comput Biol. 2014;10: e1003893 10.1371/journal.pcbi.1003893 25340343 PMC4207462

[34] Toettcher JE, Gong D, Lim WA, Weiner OD. Light-based feedback for controlling intracellular signaling dynamics. Nat Methods. 2011;8: 837–839. 10.1038/nmeth.1700 21909100 PMC3184382

[35] Milias-Argeitis A, Summers S, Stewart-Ornstein J, Zuleta I, Pincus D, El-Samad H, et al In silico feedback for in vivo regulation of a gene expression circuit. Nat Biotechnol. 2011;29: 1114–1116. 10.1038/nbt.2018 22057053 PMC4565053

[36] Menolascina F, Fiore G, Orabona E, De Stefano L, Ferry M, Hasty J, et al In-vivo real-time control of protein expression from endogenous and synthetic gene networks. PLoS Comput Biol. 2014;10: e1003625 10.1371/journal.pcbi.1003625 24831205 PMC4022480

