# Supplementary material for: What Population Reveals about Individual Cell Identity: Single-Cell Parameter Estimation of Models of Gene Expression in Yeast
Source: PLoS Comput Biol. 2016 Feb 9;12(2):e1004706. doi: 10.1371/journal.pcbi.1004706 (PMC4747589; doi:10.1371/journal.pcbi.1004706)
Supplement: S1 Text — Additional information on experimental design, data analysis, estimation of single cell quantitative features, cell lineage reconstruction, modeling of the osmostress-induced gene expression, parameter inference, simulation of population behavior, correlation with quantitative single-cell measurements, and heritability analysis. (PDF) [file pcbi.1004706.s001.pdf]

## Text S1. Supplementary Materials and Methods

### Experimental Design

In this study, we aim at characterizing the extrinsic variability in gene expression. When designing experiments, we aimed at *(i)* obtaining relatively high signal-to-noise ratio, *(ii)* obtaining highly-informative single cell traces, and *(iii)* minimizing the effect of intrinsic variability on the level of our fluorescent protein.

To obtain high signal-to-noise ratio, we used the STL1 promoter, one of the strongest osmoresponsive promoter [1], and applied hyperosmotic shocks in a repeated manner, starting experiments with a short series of 7 strong shocks. Because of cell adaptation to hyperosmotic environments, significantly higher expression levels are reached in fluctuating environments, in which stresses are repeatedly applied, than in sustained hyperosmotic environments [14].

Regarding single-cell gene expression dynamics, experiments in which periods dominated by protein production or by protein degradation are both present are in principle more informative than experiments in which only one behavior is observed. We therefore applied after the first seven shocks randomized sequences of shocks, instead of more the more conventional periodic shock profiles.

To minimize the effects of intrinsic variability on the level of our reporter protein, we used a stable fluorescent protein (yECitrine) and non-lethal but relatively strong shocks (1M sorbitol). Firstly, the use of long-lived reporters averages fast fluctuations in time. Secondly, although intrinsic variability is important for mild stresses (eg 0.1M NaCl), this effect was found to be significantly attenuated for more pronounced shocks (1). Note that care must be taken when comparing the two systems since our fluorescent reporter is integrated at the endogenous STL1 locus whereas in (1) it was inserted in the LEU2 and/or HIS3 loci and noise properties are context dependent. In particular, the endogenous locus of STL1 is very close to the telomeres of chromosome IV, a region expected to be more subjected to epigenetic effects, a potential source of extrinsic variability in gene-expression.

Lastly, we note that although the experimental design has to comply with the specific constraints of our biological system, the proposed inference approach is general.

### Data analysis

#### Datasets of single-cell gene expression measurements

The cell expression profiles were generated in three experiments: one for identification ($\mathcal{D}^I$) and two for validation ($\mathcal{D}^V$ and $\mathcal{D}^P$). In each experiment, the cells were first subjected to a series of seven hyperosmotic shocks of eight minutes every 30 minutes to obtain fluorescence measurements with good signal-to-noise ratio. After this, several osmotic shocks of eight minutes were applied with randomly-selected time intervals between shocks ($\mathcal{D}^I$ and $\mathcal{D}^V$) or with periodic shocks ($\mathcal{D}^P$). Image analysis was performed with a home-made segmentation and tracking tool, called CellStar. Raw data coming from image analysis were processed as follows. First, the data gathered from all imaging chambers were pooled together. Second, a manual review of the images and their analysis with CellStar was carried out to look for tracking problems and other possible sources of error. Missing data during the lifespan of a cell was replaced via linear interpolation when no more than one sample was missing. Subsequently, we discarded the information for the first two hours of newly detected cells after observing that usually, these cells correspond to buds that remain attached to their mother a long time after their detection. Also because of their very small size, fluorescence quantification artifacts (very dark, very steep increase) are encountered. Finally, only cells whose lifespan extended

for more than 5 hours were selected for identification and validation. The motivation for this being that cells whose lifespan is too short may not contain enough information on the dynamics of the system and may generate unreliable parameter estimates.

The datasets $\mathcal{D}^I$, $\mathcal{D}^V$, and $\mathcal{D}^P$ contain 325, 166, and 285 single-cell trajectories, respectively (see Figure S1). Among them, 63 and 39 trajectories start from time zero and remain in the device during all the experiment in $\mathcal{D}^I$ and $\mathcal{D}^V$, respectively. They are called "initial cells" and will be denoted with $\mathcal{D}^{I0}$ and $\mathcal{D}^{V0}$. Unless otherwise noted, all results are given for the identification dataset $\mathcal{D}^I$.

**Estimation of single cell quantitative features**

**Average perceived shock intensity.** Hyperosmotic shocks cause reductions of the cellular volume. The cell volume returns approximately to its pre-shock value upon restoration of the normal growth conditions. For each cell and each shock, the perceived shock intensity is defined as the relative change in volume, with the minimal and maximal volumes estimated over an 18 min time window around the considered shock. The duration of the time window was set so that it includes data before, during and after the shock. Volumes are estimated from apparent sizes in $px^2$ under the assumption that cells are spherical. Then, for each cell, the average perceived shock intensity is defined as the perceived shock intensity averaged over all shocks.

**Average cell size.** The size of each cell is computed at each time instant in bright-field images and then averaged over all time instants. Because hyperosmotic shocks lead to marked reductions of the cell volume and steep changes in the cell fluorescence, images taken less than 12 minutes after a shock are removed for this analysis.

**Average age**. The time of birth of a cell is defined as the time of its detection by the image analysis tool. It is to be noted that the cell may not yet have detached from its mother at that time. The cell's mean age is simply the average of the cell's age at every time frame. This feature cannot be defined for cells present at the beginning of the experiment (initial cells).

**Average density.** The density of the environment of a single cell is defined as the area occupied by neighbor cells relative to the area of the cell's neighborhood. The neighborhood is defined as a disk of radius of 75 px, corresponding approximately to 5 typical cell's radii. If the centroid of another cell is inside this neighborhood, then it is considered a neighbor cell. The mean density is the averaged cell's density over all time frames.

**Average division rate.** In order to automatically estimate single cell division times from bright-field images, we use the relative changes in size of cells (buds volume are not accounted for). As visible in Figure 1, these relate with budding. Since osmotic shocks greatly impact the size of cells (Figure 1), the first 12 min following shocks are discarded, yielding the dark blue curve in Figure 1 A. Two signals are defined: the first one, $s_2(t)$ (green), is obtained by smoothing twice the size curve with an 11-frame (33 min) window average; the second one, the general tendency $s_{10}(t)$ (yellow), is obtained by iteratively smoothing 10 times with an 11-frame window.

We then define the relative cell size as the relative difference between the smoothed size and the general tendency $\frac{s_2(t)-s_{10}(t)}{s_{10}(t)}$ (Figure 1 B) and compute the Fourier power spectrum of this signal. The average division time is defined as the power averaged frequency for frequencies having a period in between 60 and 400 min, conservatively including possible doubling times for yeast. This approach has been manually validated on fifty cells yielding an average error (compared to manual bud appearance based doubling rate) in the mean doubling rate of 12%.

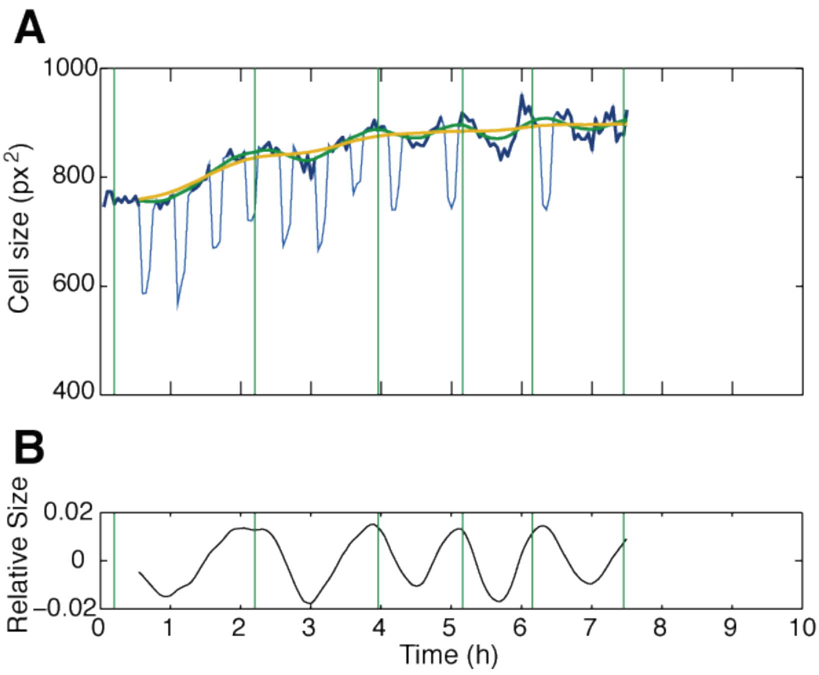


**Figure 1. Automated detection of cell budding times.** A. Plots representing the temporal evolution of the size of a cell together with several signals used for automatically extracting doubling rate. Here, the cell #6 in $\mathcal{D}^I$ was used. Light blue is the raw size. Dark blue is the raw size without shocks. Green is $s_2(t)$, the smoothed version of the size without shocks. Yellow is $s_{10}(t)$, the general tendency. Vertical green bars show manually detected bud appearance time (shown for validation purpose). B. Normalized cell size used for the Fourier analysis. Vertical green bar are the same as in A.

### Cell lineage reconstruction

After automated segmentation and tracking, lineage was manually extracted from microscopy images for the first imaging chamber of the identification experiment $\mathcal{D}^I$ in order to retrieve the complete lineage tree for the cells tracked in that chamber. Among the 86 cells in the chambers, 55 mother-daughter relationships are identified (the experiment starts with 26 cells in the fields of view and 5 additional cells come from the outside).

## Model of osmostress-induced gene expression

### Gene expression model

We use here the following model of gene expression:

$$\begin{cases} \dot{m}(t) = k_m u(t) - g_m m(t), \\ \dot{p}(t) = k_p m(t) - g_p p(t), \end{cases}$$

where $m$ and $p$ denote, respectively, the cellular concentration of the mRNA and of the fluorescent protein yECitrine. Synthesis and degradation rates for mRNA are represented by $k_m$ and $g_m$, whereas their respective counterparts for the protein are denoted with $k_p$ and $g_p$. At time zero, we consider the initial concentrations $m_0 = p_0 = 0$. The input function $u(t)$ represents the phosphorylation and nuclear import of the Hog1 protein, and like in Uhlendorf *et al* (2012) [14] and Muzzey *et al* (2009), we assume that it depends on the osmolarity effectively sensed by the cells inside the microfluidic chambers $u_c(t)$ as follows:

$$\dot{u}(t) = k_h u_c(t) - g_h u(t).$$

In accordance with the observations made in Zechner et al (2012) [5], we assume that in comparison to gene expression, signal transduction shows little variability. Therefore, we assume fixed values for $k_h$

and $g_h$: $k_h = 0.3968$ and $g_h = 0.9225$ (3). Lastly, as shown in Uhlendorf *et al* (2012) [14], there is a known lag between the valve actuation $u_v(t)$ and the actual change in the osmolarity of the cellular environment in the imaging chamber $u_c(t)$. This relation can be simply represented by a piecewise linear function. The relations between the valve status, the chamber osmolarity, and the Hog1 activity are graphically represented in Figure 2 for an 8-minute shock.

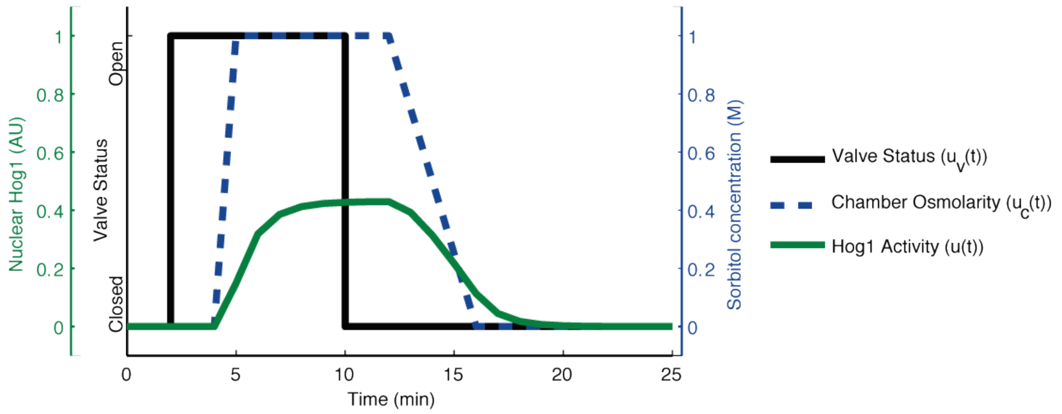


**Figure 2: Temporal evolution of the osmolarity of the cellular environment $u_c$, and of the Hog1 activity $u$, as a function of the position of the microfluidic valve $u_v$ (0/1: normal/hyper-osmotic medium).**

In order to account for fluorescent protein maturation time, we introduce a delay $\tau$. The measured concentration of mature protein depends on the total (mature or not) protein concentration $\tau$ instants before and on the dilution rate due to cell (exponential) growth (we neglect degradation and photobleaching, see section Initial parameter values). To establish these relations, consider a cell that grows at a rate $g_p$. Then, $p(t)$ is the total protein concentration at time $t$, and we denote $P(t)$, $F(t)$, $V(t)$, and $f(t)$, the total and mature protein amounts, the cell volume, and the mature protein concentration, respectively. Then it holds that $V(t) = V(t-\tau)e^{g_p\tau}$ and that $F(t) = P(t-\tau)$. So, we can describe the cell fluorescence as

$$f(t) = \frac{F(t)}{V(t)} = \frac{P(t-\tau)}{V(t-\tau)e^{g_p\tau}} = e^{-g_p\tau}p(t-\tau).$$

We assume $p(t) = 0$ for $t \leq 0$. The choice of representing protein maturation by a delay rather than with first or second order linear reaction comes from the observation that in our data there is a delay following a shock during which absolutely no increase in fluorescence is observed. Finally, we assume a Gaussian noise model for the observations noise, with an additive component and a multiplicative component, meaning that the measured cell fluorescence $y(t)$ follows

$$y(t) = f(t) + h(t)\eta(t),$$

with $h(t) = (\varepsilon_a + f(t)\varepsilon_b)$, and where $\eta(t)$ is white Gaussian noise with mean 0 and intensity 1, and ${\varepsilon_a}^2$, ${\varepsilon_b}^2$ define the intensity of the additive and multiplicative noise components. NB: experimental noise being considered iid, this formulation is equivalent to having two independent Gaussian white noises for the additive and multiplicative contributions by additivity of Gaussian random variables. Also in the SAEM inference these noise parameters are the same for all the cells. For the Naïve inference, it is not possible to infer noise parameters shared by all cells. These have either to be fixed or to be estimated separately for each cell. Nevertheless, estimating measurement noise parameters for each cell gives very close noise values to that found with SAEM. We verified that fixing the same measurement noise for the Naïve approach did not change the results.

**Mixed-effects model of gene expression**

In this framework, we assume that cells share the structural model of gene expression described above but that their parameters are different. Let denote $S$ the number of molecular species, and $x(t) = [x_1(t), \ldots, x_S(t)]^T$ the vector of their respective concentrations at time $t$. The velocity of the change in concentration for each species can be described as a differential equation of the form

$$\dot{x}_i(\mathrm{t}) = v(\mathrm{x(t)}, u(t), \psi_i)$$

where $i = 1, \ldots, \mathrm{N}$ and N is the total number of cells and $\psi_i$ are cell-specific parameters. System quantities that are measured over time can be described via an output equation:

$$y_i(\mathrm{t}) = \tilde{v}(x_i) + h(\tilde{v}(x_i), \xi)\eta_i(t)$$

where the vector $y_i(\mathrm{t}) \in \mathrm{R^m}$ is the system output. Function $\tilde{v}(\cdot)$ allows us to select state variables that are observed over time (e.g. total concentration of protein). We assume output measurements are corrupted by additive and multiplicative noise, i.e.

$$h(\tilde{v}(x_i), \xi) = (\varepsilon_a + \tilde{v}(x_i)\varepsilon_b)$$

$\eta_i$ represents white Gaussian noise where $\eta_i(t) \sim N(0,1)$. Denoting the output transition map when $\eta_i(t) = 0$ as $f(t, \mathrm{u}, x_0, \psi_i)$, we have:

$$y_i(t) = f(t, u, x_0, \psi_i) + h(t, u, x_0, \psi_i, \xi)\eta_i(t)$$

For a given observation at time $t_j$ :

$$y_{ij} = f(t_j, \mathrm{u}, x_0, \psi_i) + h(t_j, \mathrm{u}, x_0, \psi_i, \xi)\eta_{ij}, \qquad i = 1, \ldots, N \;\; j = 1, \ldots, T$$

This equation is called the individual-level model.

For our particular system, $x_0 = x(t_0) = \{m_0, p_0\}$, $\psi_i = \{k_{mi}, g_{mi}, k_{pi}, g_{pi}, \tau\}$, $\xi = \{\varepsilon_a, \varepsilon_b\}$, and:

$$v(\mathrm{x(t)}, u(t), \psi_i) = \begin{cases} \dot{m}(t) = k_m u(t) - g_m m(t), \\ \dot{p}(t) = k_p m(t) - g_p p(t), \end{cases}$$

$$\tilde{v}(x_i) = e^{-g_p \tau} p(t - \tau)$$

Note that the noise parameters $\xi = \{\varepsilon_a, \varepsilon_b\}$ are constant over the whole population of cells.

The single-cell parameters $\psi_i$ depend themselves on a model that defines their statistics. This model is called population-level model and it defined as

$$\psi_i = d(\mu, b_i); \; b_i \sim N(0, \Omega)$$

where $\mu$ is called the vector of fixed-effects and represents the typical parameters of the population, which are not to be confused with the mean-cell parameters. Vector $b_i$ denotes the random effects, drawn from a multivariate Gaussian distribution with mean 0 and covariance matrix $\Omega$, which determine how different from $\mu$ the individual parameters are. Function $d(\cdot)$ performs a monotonic transformation on the parameters to have non-normal parameter distributions. Here we assumed that the parameters $\psi_i$ are log-normally distributed:

$$\psi_i = d(\mu, b_i) = e^{\mu + b_i}, \text{with } \varphi_i = \mu + b_i \text{ and } \varphi_i \sim N(\mu, \Omega)$$

where for a vector $\varphi_i = [\varphi_{i_1}, \ldots, \varphi_{i_P}]$, we define the element-wise operation $e^{\varphi_i} = [e^{\varphi_{i1}}, \ldots, e^{\varphi_{ip}}]$. The set of parameters to identify is $\theta = \{\mu, \Omega, \xi\}$. Note that the number of parameters to identify scales quadratically with the number of model parameters.

## Parameter inference

### Initial parameter values

Initial parameter values are estimated based on literature data as described in the table below.

| Parameter | Description | Unit | Reference value | Reference value (Log. scale) | Source |
|---|---|---|---|---|---|
| $k_m$ | Transcription rate | $min^{-1}$ | $1.00\ 10^1$ | 2.30 | [26] |

| | | | | | |
|---|---|---|---|---|---|
| $g_m$ | mRNA degradation rate | $min^{-1}$ | $2.94\ 10^{-1}$ | -1.22 | [26][1] |
| $k_p$ | Translation rate | $min^{-1}$ | $9.47\ 10^{-1}$ | $-5.4\ 10^{-2}$ | Computations using (4) & (5) [2] |
| $g_p$ | Protein decay rate | $min^{-1}$ | $4.00\ 10^{-3}$ | -5.52 | This study [3] |
| $\tau$ | Protein maturation time | min | $3.00\ 10^{1}$ | 3.40 | This study [4] |

[1] Note that the value used as reference taken from [26] appeared later to be inappropriate because we express here an exogenous mRNA (pSTL1-yECitrine) which differs on the 3' end from that used in [26]. A more conservative value would be to take the average mRNA degradation rate in yeast (1,31 $10^{-2}$) as measured in (6). Indeed, we found an estimated value closer to this average value and similar to that of native STL1 transcripts as measured in [21].

[2] Translation rate was estimated by using the rate of translation initiation and the rate of correct translation using models from (4, 5) calibrated for our specific DNA sequence.

[3] This term comes from three processes: degradation, photo-bleaching and dilution. Degradation was assumed to be negligible since yECitrine is very stable. The dilution rate was computed from the average doubling rates estimated on $\mathcal{D}^I$ and $\mathcal{D}^V$ (linear fit on the logarithmic number of cells). It yields an average dilution rate of 4 $10^{-3}$ $min^{-1}$. Photo-bleaching was estimated by repeatedly imaging a cell population and extracting a bleaching rate per frame by fitting the decay curve obtained. This yields a bleaching rate of 3.5 $10^{-4}$ $min^{-1}$.

[4] The value is based on the time required for the cells to respond to the first stimulus. Note that this value was larger for $\mathcal{D}^V$.

**Inference of mixed-effects model: the naive approach**

One intuitive approach for estimating single-cell and population parameters in a mixed-effects framework consists in obtaining individual estimates of rate parameters $\psi_i = \left\{k_{m_i}, g_{m_i}, k_{p_i}, g_{p_i}, \tau_i\right\}$ and noise parameters $\xi_i = \{\varepsilon_{a_i}, \varepsilon_{b_i}\}$ by fitting one cell at a time and then to compute the population statistics directly from the set of obtained parameters. For each cell $i = 1, \dots, N$, the total set of parameters $\theta_i = \{\psi_i, \xi_i\}$ has been inferred using maximum likelihood estimation (MLE), yielding a total of $7 \times N$ inferred parameters. Given that the estimation of the single-cell parameters, $\theta_i$, is done cell by cell, one has to solve $N$ optimization problems, each involving 7 variables only. This is done as follows:

$$\mathcal{L}(\theta_i|Y_i) = p(Y_i|\theta_i)$$

$$Y_{ij} = f(t_j, \mathrm{u}, x_0, \psi_i) + \varepsilon_j \qquad \varepsilon_j \sim N\left(0, \left(h(t_j, \mathrm{u}, x_0, \psi_i, \xi_i)\right)^2\right)$$

So, for a given sample in cell $i$ at time $j$:

$$p(Y_{i,j}|\theta_i) = \frac{1}{\sqrt{2\pi} \cdot h\left(f(t_j, \mathrm{u}, x_0, \psi_i), \xi_i\right)} \exp\left(-\frac{1}{2}\left(\frac{Y_{ij} - f(t_j, \mathrm{u}, x_0, \psi_i)}{h\left(f(t_j, \mathrm{u}, x_0, \psi_i), \xi_i\right)}\right)^2\right)$$

$$\log[p(Y_{i,j}|\theta_i)] = \log\frac{1}{\sqrt{2\pi}} - \log\left(h\left(f(t_j, \mathrm{u}, x_0, \psi_i), \xi_i\right)\right) - \frac{1}{2}\left(\frac{Y_{ij} - f(t_j, \mathrm{u}, x_0, \psi_i)}{h\left(f(t_j, \mathrm{u}, x_0, \psi_i), \xi_i\right)}\right)^2$$

And, for the complete set of samples in cell $i$:

$$p(Y_i|\theta_i) = p(Y_{i,1}, Y_{i,2}, \dots, Y_{i,j}|\theta_i) = \prod_j p(Y_{i,j}|\theta_i)$$

Therefore:

$$\log[p(Y_i|\theta_i)] = -\sum_j \frac{1}{2}\left(\frac{Y_{ij} - f(t_j, \mathrm{u}, x_0, \psi_i)}{h\left(f(t_j, \mathrm{u}, x_0, \psi_i), \xi_i\right)}\right)^2 - \sum_j \log\left(h\left(f(t_j, \mathrm{u}, x_0, \psi_i), \xi_i\right)\right) + \sum_j \log\frac{1}{\sqrt{2\pi}}$$

The last term is a constant, thus can be removed from the equation. Now we can compute:

$$\hat{\theta}_{i_{ML}} = \underset{\psi_i, \xi_i}{\text{Argmax}} \left[ -\frac{1}{2} \sum_j \left( \frac{Y_{ij} - f(t_j, \mathrm{u}, x_0, \psi_i)}{h(f(t_j, \mathrm{u}, x_0, \psi_i), \xi_i)} \right)^2 - \sum_j \log\left( h(f(t_j, \mathrm{u}, x_0, \psi_i), \xi_i) \right) \right]$$

The maximization is performed using the *fminsearch* function from Matlab. For convenience, all the parameters are estimated in the logarithmic scale, which is equal to estimating the normally-distributed variables $\varphi_i$ as defined in the mixed-effects model section. Denoting $\varphi = \{\varphi_1, \varphi_{2,} \dots \varphi_N\}^T$, the population statistics $\mu, \Omega$ and $\xi$ are computed in the following way.

$$\mu = \frac{1}{N} \sum_{i=1}^{N} \varphi_i\,, \qquad \Omega = \frac{1}{N-1} \sum_{i=1}^{N} (\varphi_i - \mu)(\varphi_i - \mu)^T\,, \qquad \xi = \frac{1}{N} \sum_{i=1}^{N} \xi_i$$

**Inference of mixed-effects model: the SAEM approach**

Instead of the naive approach, one can choose a more inclusive approach, in which we directly estimate the population statistics by accounting simultaneously for the ensemble of all cell's observations. Single-cell fits can be computed a posteriori using the population distribution as prior knowledge (see below). The estimation of the population statistics can be done via population-likelihood maximization algorithms, such as the Stochastic Approximation Expectation Maximization (SAEM) [11]. Here the set of parameters to identify is $\theta = \{\mu, \Omega, \xi\}$. At each iteration of the algorithm, the objective is to maximize the log-likelihood [Lixoft, Monolix Methodology v.4.3.3, 2014]

$$\log p(Y, \psi\,|\theta) = -\sum_{i,j} \log\left( h(x_{ij}, \psi_i, \xi) \right) - \frac{1}{2} \sum_{i,j} \left( \frac{y_{ij} - f(x_{ij}, \psi_i)}{h(x_{ij}, \psi_i, \xi)} \right)^2 - \frac{N}{2} \log(|\Omega|)$$

$$- \frac{1}{2} \sum_{i=1}^{N} (\varphi_i - \mu)' \Omega^{-1} (\varphi_i - \mu) - \frac{N_{tot} + N_d}{2} \log(2\pi),$$

where $N_{tot}$ is the total number of samples and $N_d$ is the number of degrees of freedom. We used the SAEM implementation available in the Monolix software [20].

**Inference of single-cell models from population models: a MAP approach**

Based on a population distribution with parameters $\theta = \{\mu, \Omega, \xi\}$, single cell estimates $\psi_i$ are obtained via maximum *a posteriori* estimation (MAP).

$$\hat{\psi}_{i_{MAP}} = \underset{\psi_i}{\text{Argmax}} [p(\psi_i | Y_i, \mu, \Omega, \xi)] = \underset{\psi_i}{\text{Argmax}} \left[ \frac{p(Y_i | \psi_i, \xi) \cdot p(\psi_i | \mu, \Omega)}{p(Y_i | \mu, \Omega, \xi)} \right]$$

Note that, because of identifiability issues, $\psi_i$ might be reduced to 4 effective parameters only, where $k_{mp}$ captures the product of $k_m$ and $k_p$ (see main text and Text S4). By a slight abuse of notation, and because it should be clear from the context, we use $\psi_i$ to denote both vectors. As $p(Y_i | \mu, \Omega, \xi)$ does not depend on $\psi_i$, it can be removed from the equation. Then:

$$\hat{\psi}_{i_{MAP}} = \underset{\psi_i}{\text{Argmax}} [\log[p(Y_i | \psi_i, \xi)] + \log[p(\psi_i | \mu, \Omega)]]$$

The first term, $\log[p(Y_i | \psi_i, \xi)]$, corresponds to the log-likelihood $\log[p(Y_{i,j} | \theta_i)]$ explained previously summed over $j$ (the only difference being that in the present case the noise parameters $\xi$ are common to all cells. For the second term, $\log[p(\psi_i | \mu, \Omega)]$, we have:

$$p(\psi_i | \mu, \Omega) = \frac{1}{\sqrt{2\pi^n |\Omega|}} \exp\left( -\frac{1}{2} (\psi_i - \mu)^T \Omega^{-1} (\psi_i - \mu) \right)$$

$$\log[p(\psi_i|\mu,\Omega)] = \log\left(\frac{1}{\sqrt{2\pi^n|\Omega|}}\right) - \frac{1}{2}(\psi_i-\mu)^T\Omega^{-1}(\psi_i-\mu)$$

The term $\log\left(\frac{1}{\sqrt{2\pi^n|\Omega|}}\right)$ being a constant, we obtain:

$$\hat{\psi}_{i_{MAP}} = \underset{\psi_i}{\text{Argmax}}\left[-\frac{1}{2}\sum_j\left(\frac{Y_{ij}-f(t_j,\mathrm{u},x_0,\psi_i)}{h(f(t_j,\mathrm{u},x_0,\psi_i),\xi)}\right)^2 - \sum_j \log\left(h(f(t_j,\mathrm{u},x_0,\psi_i),\xi)\right) - \frac{1}{2}(\psi_i-\mu)^T\Omega^{-1}(\psi_i-\mu)\right]$$

## Simulation of population behavior

Predictions of the behavior of cell populations using mixed-effects models are obtained by sampling 10000 parameter values from the distributions and performing the corresponding numerical simulations.

## Correlation with quantitative single cell measurements

To compute correlations between single cell features and estimated parameters, we used the rank-based Spearman coefficient of variation. The standard two-tailed statistical test included in the Matlab statistics toolbox is used to test the significance of the correlations (p-values). Note that a few cells have been discarded because a given feature cannot always be determined for those cells. For instance, cells present at the beginning of the experiment cannot be assigned an age.

## Heritability analysis

We wanted to test whether single cell parameter values could capture some form to inheritance. We considered the average mother-daughter distance in parameter values using the Euclidian distance $d(q_i, q_j) = \sqrt{(q_j - q_i)^2}$. This distance, computed parameter by parameter, was then compared to the distribution of parameter distances for random pairs taken from the whole experiment population, and showed significant differences (Figure S4). Nevertheless, this comparison is subjected to several biases, mainly that mother and daughter parameters could be closer only because they share a more similar environment. To compensate for these biases, the average mother-daughter parameter value was compared to that of a more thoughtfully constructed control population. First, we only considered cells coming from the same chamber. Second, from this original set of mother-daughter pairs, we constructed a control set of pairs of cells where every pair is made of one mother cell and one daughter cell of a different mother. This allows comparing two set of pairs of cells (related mothers and daughters, termed MD and non-related mothers and daughters, termed nMD) which are made from exactly the same cells and only differ by the presence of direct lineage relationship, therefore minimizing the previously mentioned biases. The MD set is made of 55 pairs and the nMD set is made of 1870 pairs. We see that mean distances based on MD pairs are always smaller than mean distances based on nMD pairs. Nevertheless, we wanted to derive a p-value on this hypothesis. Because the distribution of distances between pairs of parameters is a priori of unknown shape (and in practice non-gaussian), bootstrapping, a standard method to derive confidence intervals for random variables with unknown distribution was used. *p*-values are based on the Welch two sided t-test for checking whether the mean of 40 cells in MD is smaller than the mean of 40 cells in nMD. The effective degree of freedom was computed using the Welch-Satterthwaite equation. In the main text example, we used

50 000 bootstrapped sets of 40 pairs. Similar results were obtained when we used a different number of bootstrapped sets, and smaller and bigger sets (*e.g.* bootstrapping sets of 5, 10, 20, 30, 40 and 50 cells).

## Bibliography


1. Pelet S, et al. (2011) Transient Activation of the HOG MAPK Pathway Regulates Bimodal Gene Expression. *Science* 332(6030):732–735.

2. Muzzey D, Gómez-Uribe C, Mettetal JT, van Oudenaarden A (2009) A systems-level analysis of perfect adaptation in yeast osmoregulation. *Cell* 138(1):160–71.

3. Uhlendorf J, Bottani S, Fages F, Hersen P, Batt G (2011) Towards real-time control of gene expression: controlling the HOG signaling cascade. *Pacific Symposium on Biocomputing*, pp 338–49.

4. Shah P, Ding Y, Niemczyk M, Kudla G, Plotkin JB (2013) Rate-limiting steps in yeast protein translation. *Cell* 153(7):1589–601.

5. Gilchrist M, Wagner A (2006) A model of protein translation including codon bias, nonsense errors, and ribosome recycling. *J Theor Biol* 239:417–434.

6. Wang Y, et al. (2002) Precision and functional specificity in mRNA decay. *Proc Natl Acad Sci U S A* 99(9):5860–5865.
