# Supplementary material for: What Population Reveals about Individual Cell Identity: Single-Cell Parameter Estimation of Models of Gene Expression in Yeast
Source: PLoS Comput Biol. 2016 Feb 9;12(2):e1004706. doi: 10.1371/journal.pcbi.1004706 (PMC4747589; doi:10.1371/journal.pcbi.1004706)
Supplement: S2 Text — Influence of the cell number and of the learning time horizon. (PDF) [file pcbi.1004706.s002.pdf]

## Text S2. Robustness of population predictions: influence of the cell number and of the learning time horizon

### Influence of cell number on the robustness of population predictions

What is the minimum number of cells we have to track in order to obtain reliable estimates? This is an important question to address when dealing with the identification of parameter distributions. We therefore tested the robustness of SAEM inference with respect to the number of cells available in the identification dataset for ME models. For this purpose we repeatedly estimated ME models from datasets containing only a few cells and quantified the variability of the corresponding predictions. More precisely, we extracted from $\mathcal{D}^{I0}$ 25 sub-sets $\mathcal{D}^{I0}_{c,n}$ with $c = 2,4,8,16,32$ and $n = 1,..,5$. Each subset has a number $c$ of cells which will be extracted randomly $n$ times from dataset $\mathcal{D}^{I0}$. We performed population parameter inference in each subset, and compared the quantiles of the predicted population to those of the observed population. The selected quantiles were q=0.5, q=0.025 and q=0.975. These values represent, respectively, the median of the population and the lower and higher bound of the 95% of the population. For the comparison, we used the root mean squared error, normalized by the difference between the maximal and minimal observed values (NRMSE). Table 1 shows the computed NRMSE values for each test and each quantile. The means and standard deviations of the NRMSE in the different tests are indicated in Table 1 and graphically represented in Figure 1. They give a measure of the accuracy and the uncertainty of the estimates. The uncertainty is large when there are only two cells (the quantile's predictions even overlap), but rapidly decreases and stabilizes above 16 cells.

**Table 1. Effect of the number of cell traces on the robustness of the predictions.** The deviation (NRMSE) between the predicted quantiles and the observed quantiles for 5 random subsets of cells is reported. The mean is an indicator of the accuracy of the prediction. The standard deviation is an indicator of the dispersion of these predictions; a low SD indicates that the predictions do not vary considerably when selecting different subsets with the given number of cells.

| Robustness of population predictions with respect to number of cells in $D^I$ | | | | | | | | |
|---|---|---|---|---|---|---|---|---|
| | | NRMSE(q) | | | | | | |
| **# of Cells** | **Quantile** | **Test 1** | **Test 2** | **Test 3** | **Test 4** | **Test 5** | **Mean** | **SD** |
| **2** | **q0.025** | 0.14 | 0.37 | 0.33 | 0.32 | 0.14 | **0.26** | **0.11** |
| | **q0.5** | 0.11 | 0.13 | 0.22 | 0.08 | 0.07 | **0.12** | **0.06** |
| | **q0.975** | 0.08 | 0.05 | 0.16 | 0.07 | 0.10 | **0.09** | **0.04** |
| **4** | **q0.025** | 0.15 | 0.20 | 0.11 | 0.06 | 0.13 | **0.13** | **0.05** |
| | **q0.5** | 0.06 | 0.06 | 0.06 | 0.08 | 0.07 | **0.07** | **0.01** |
| | **q0.975** | 0.08 | 0.08 | 0.06 | 0.13 | 0.07 | **0.08** | **0.03** |
| **8** | **q0.025** | 0.09 | 0.11 | 0.05 | 0.11 | 0.09 | **0.09** | **0.02** |
| | **q0.5** | 0.06 | 0.06 | 0.07 | 0.06 | 0.05 | **0.06** | **0.01** |
| | **q0.975** | 0.12 | 0.05 | 0.12 | 0.07 | 0.10 | **0.09** | **0.03** |
| **16** | **q0.025** | 0.05 | 0.06 | 0.14 | 0.07 | 0.07 | **0.08** | **0.04** |
| | **q0.5** | 0.06 | 0.06 | 0.05 | 0.06 | 0.07 | **0.06** | **0.01** |
| | **q0.975** | 0.10 | 0.07 | 0.08 | 0.09 | 0.09 | **0.09** | **0.01** |
| **32** | **q0.025** | 0.06 | 0.06 | 0.05 | 0.06 | 0.07 | **0.06** | **0.01** |
| | **q0.5** | 0.06 | 0.05 | 0.06 | 0.06 | 0.06 | **0.06** | **0.00** |
| | **q0.975** | 0.10 | 0.06 | 0.09 | 0.12 | 0.08 | **0.09** | **0.02** |

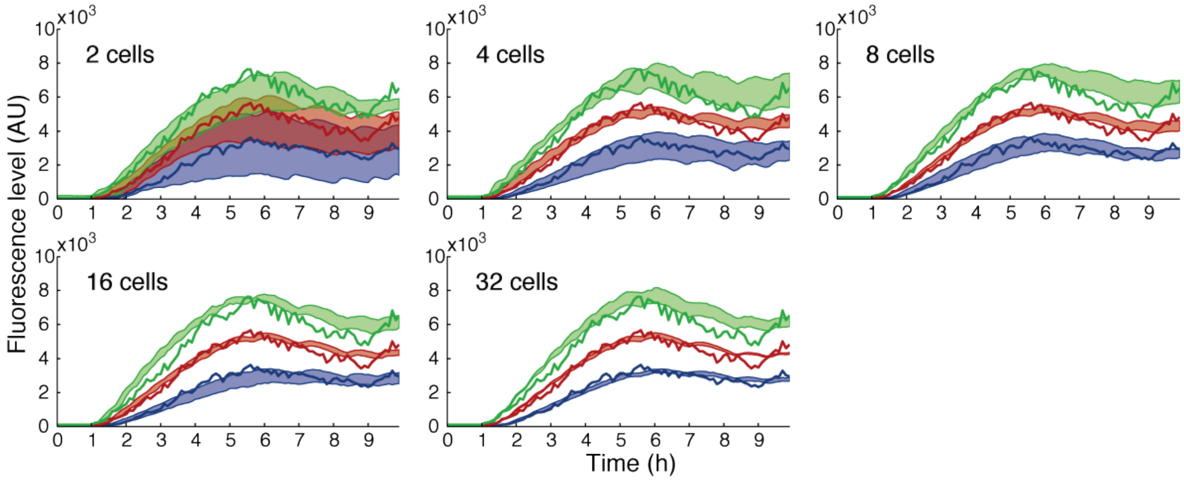


**Figure 1. Effect of the number of cell traces on the robustness of the predictions.** Green, red and blue solid lines denote, respectively the 0.025, 0.5 and 0.975 quantiles, corresponding to 95% of the observed population in $\boldsymbol{D^I}$ (325 cells). The shaded areas with the corresponding colors represent the maximum and minimum boundaries of the quantiles estimated with 5 randomly selected subsets of 2,4,8,16 and 32 cells. Thinner shaded areas indicate less variability in the predictions. After 16 cells the width of these areas has decreased considerably.

## Influence of the learning time horizon on the robustness of population predictions

We tested the robustness of SAEM inference with respect to the duration of the learning time horizon (observation time $T_{obs}$) by testing the prediction capabilities of the resulting mixed-effect models on the rest of the data (prediction time $T_{pred}$). We used the identification dataset $D^I$. ME models inferred on datasets with 5 or 6 hours of observations show bad prediction capabilities on the subsequent hours. After 7 hours the performance increases significantly (Figure 2). This suggests that an accurate inference of the model's parameter values in this experimental setup requires acquisition of data during extended time intervals.

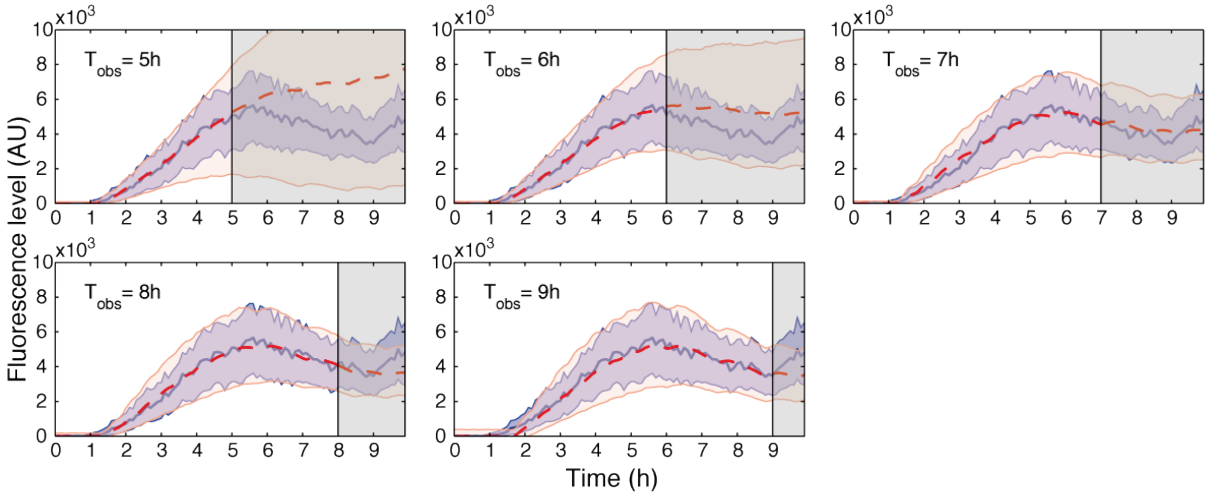


**Figure 2. Influence of the learning time horizon in population predictions.** The blue line and blue shaded area represent observed cell populations (median and 95% of the population). Red dashed line and pink shaded area represent the model predictions during observation time ($T_{obs}$) and prediction time ($T_{pred}$, gray shaded area) (again median and 95% of the population). All are given for experiment $\boldsymbol{D^I}$.
