# Supplementary material for: What Population Reveals about Individual Cell Identity: Single-Cell Parameter Estimation of Models of Gene Expression in Yeast
Source: PLoS Comput Biol. 2016 Feb 9;12(2):e1004706. doi: 10.1371/journal.pcbi.1004706 (PMC4747589; doi:10.1371/journal.pcbi.1004706)
Supplement: S3 Text — Predicting population behavior on two validation data sets. (PDF) [file pcbi.1004706.s003.pdf]

Text S3. Validation of population predictions: predicting population behavior on two validation data sets

To test the capacity of our model and inferred parameter distribution to predict the behavior of cell populations, we generated two validation datasets. The first one, $\mathcal{D}^V$, uses temporal profile of hyperosmotic shock that is different from but close to the identification dataset $\mathcal{D}^I$. The second one, $\mathcal{D}^P$, uses periodic shocks (8 minutes shocks every half-hour) and is markedly different from the identification dataset.

In both cases, we simulated 1000 single-cell traces using the population distribution of parameters estimated on $\mathcal{D}^I$. Prediction results are represented on Figure 1. On $\mathcal{D}^V$, the prediction quality was acceptable (Fig 1A). However, a significant bias was observed for the validation dataset $\mathcal{D}^P$ (Fig 1B – see the difference between real and predicted median profiles). How can this be explained? One of the results of our study is that, in addition to hyperosmotic shocks, several factors are likely to influence gene expression, one of them being the cell division rate. And indeed, cells grow and divide in $\mathcal{D}^P$ significantly slower than in $\mathcal{D}^I$ (mean division rates are 3.5 10^{-3} and 6.3 10^{-3} min^{-1}, respectively). This growth rate difference can be explained by the significantly higher total amount of stress imposed to cells in $\mathcal{D}^P$.

Because protein degradation rate and photobleaching can be neglected in comparison to the dilution effect due to growth (see section Initial parameter values), one can correct the parameter g_p in a systematic manner. In fact, when replacing the median value of g_p from the population distribution of parameters estimated on $\mathcal{D}^I$ with the empirical population division rate, the prediction capability improves and the systematic bias is effectively corrected (Fig 1C). Yet, we observe that the predicted variability is still somewhat lower than the observed one. This might likely come from other differences in environmental or physiological conditions. Unlike the possible correction of g_p which used a direct relationship between a measurable influence factor (division rate) and a parameter of our model, most other influence factors cannot be similarly corrected for prediction purposes. This is because either they cannot be measured, or because applying a correction would require a specific model of the relationship between these factors and single cell parameter values.

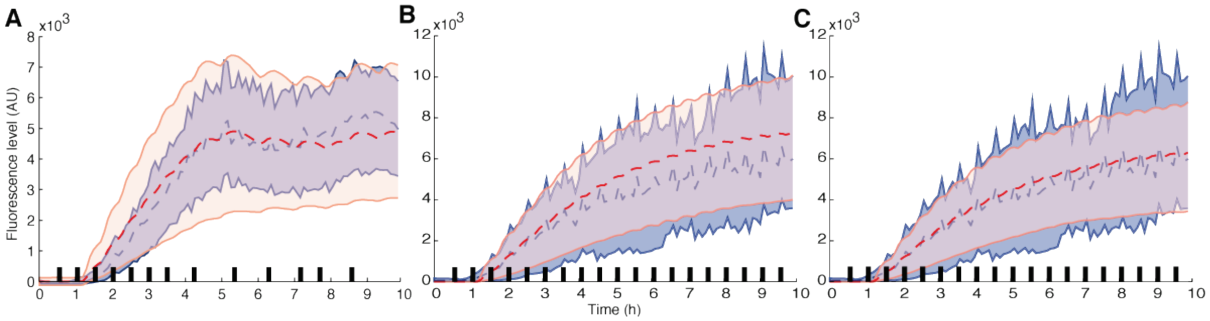

Figure 1. Predicting population behavior on validation data sets (A) Prediction for experiment $\mathcal{D}^V$ using a population model estimated on $\mathcal{D}^I$. (B) Prediction for experiment $\mathcal{D}^P$ using a population model estimated on $\mathcal{D}^I$. (C) Prediction for experiment $\mathcal{D}^P$ using a population model estimated on $\mathcal{D}^I$ for which the average dilution rate g_p was set to the population division rate measured in $\mathcal{D}^P$. Shaded areas represent the fluorescence values of 95% of the population and the dashed lines represent the median. Experimental data is represented in blue and simulations of 1000 virtual cells are shown in pink. Black bars indicate the presence of osmotic shocks. Note the different scale for the y-axis.
