# Supplementary material for: What Population Reveals about Individual Cell Identity: Single-Cell Parameter Estimation of Models of Gene Expression in Yeast
Source: PLoS Comput Biol. 2016 Feb 9;12(2):e1004706. doi: 10.1371/journal.pcbi.1004706 (PMC4747589; doi:10.1371/journal.pcbi.1004706)
Supplement: S4 Text — Parameters km and kp cannot be assigned values unambiguously no matter the quality and quantity of fluorescence measurements. (PDF) [file pcbi.1004706.s004.pdf]

Text S4. Identifiability analysis

We show here that parameters k_m and k_p cannot be assigned values unambiguously no matter the quality and quantity of fluorescence measurements. Since the gene expression model giving mRNA (m) and protein (p) levels as a function of time is a linear dynamical system, and the cell fluorescence f is assumed to be simply a rescaled and delayed version of p, we can easily verify this by looking at the transfer function of the system (1). This is given by

$$F(s) = e^{-\tau(g_p+s)}P(s),$$

$$P(s) = \frac{k_m\ k_p}{(s + g_m)(s + g_p)}U(s) + \frac{k_p}{(s + g_m)(s + g_p)}m(0) + \frac{1}{s + g_p}p(0),$$

where $U(s)$, $P(s)$ and $F(s)$ are the Laplace transforms of $u(t)$, $p(t)$ and $f(t)$, in the same order. For any fixed input $U(s)$, since $m(0) = p(0) = 0$, it is apparent that $F(s)$ depends on k_m and k_p only via their product. That is, all models with the same values of g_m, g_p and $k_m \cdot k_p$ will respond identically to the same input no matter the specific values of k_m and k_p. A similar issue would arise if $m(0)$ was different from zero but unknown. In this case, the term depending on k_p only would actually depend on the product $k_p m(0)$, with both factors unknown. This issue is commonly referred to as "structural non-identifiability'' (of k_m and k_p).

Structural non-identifiability of k_m and k_p generally results in issues in the identification of their population statistics as well. In order to ensure a well-posed mixed-effects identification problem, all identification results reported in this work were obtained with the mean of k_p fixed to a default value. We stress the fact that, although related, single-cell model (non-)identifiability should not be confused with the (non-)identifiability of the parameter statistics in the mixed-effects approach. To what extent, if at all, identifiability of the statistics of non-identifiable single-cell parameters is ameliorated by a population approach (e.g. through their correlation with yet other parameters) is not obvious. While a full theoretical investigation of this issue would go beyond the scope of this paper, this point is illustrated on a simple example, analogous to our case study, in Text S5.

When using Mixed Effects models and SAEM, controlling shrinkage is also useful in order to detect potential identifiability-related issues. We speak of shrinkage when the empirical distribution of single-cell parameters (as estimated by MAP or maximum likelihood) is narrower than the population distribution. Obtaining shrinkage is indeed reminiscent of having single-cell parameters ill-defined (having a flat likelihood). In such a case, single-cell parameter estimates given by MAP will mostly represent the mode of the prior (i.e. the population distribution), resulting in a narrow distribution of single-cell parameter values (2). Subsequently to our non-identifiability analysis, we found that no substantial shrinkage was present. Indeed, computing the η-shrinkage as in (2) yielded 12%, 0% and 4% for parameters k_{mp}, g_p and g_m on $\mathcal{D}^I$, and 6%, 4% and 4% on $\mathcal{D}^V$, respectively.

Bibliography

1. Oppenheim AV., Willsky AS, Nawab SH (1996) Signals and Systems. Prentice Hall
2. Savic RM, Karlsson MO (2009) Importance of shrinkage in empirical Bayes estimates for diagnostics: problems and solutions. AAPS Journal, 11(3):558–569
