# Supplementary material for: What Population Reveals about Individual Cell Identity: Single-Cell Parameter Estimation of Models of Gene Expression in Yeast
Source: PLoS Comput Biol. 2016 Feb 9;12(2):e1004706. doi: 10.1371/journal.pcbi.1004706 (PMC4747589; doi:10.1371/journal.pcbi.1004706)
Supplement: S5 Text — Statistical properties of parameters that are not distinguishable at the single-cell level can nevertheless be constrained in a population approach. (PDF) [file pcbi.1004706.s005.pdf]

Text S5. On learning the statistics of non-identifiable parameters

We illustrate here that statistical properties of parameters that are not distinguishable at the single-cell level can nevertheless be constrained in a population approach. To this aim, we consider a simple model, linear in its parameters:

$$\begin{aligned} y_1 &= \theta_1 + \theta_2 \\ y_2 &= \theta_3 \end{aligned}$$

In this model, θ_1 and θ_2 cannot be distinguished from one observation of the outputs, i.e. they would be non-identifiable at the single-cell level. Note that the situation is analogous to the one encountered in our gene expression model considering log-transformed parameters. Indeed $\log(k_m)$ and $\log(k_p)$ are reflected in the model output only via their sum, $\log(k_{mp})$ (see Text S3 Identifiability analysis). Denoting the experimentally observable vector variable $y = [y_1, y_2]^T$, and the vector of parameters $\theta = [\theta_1 \dots \theta_3]^T$, we have

$$y = L\theta, \text{with } L = \begin{bmatrix} 1 & 1 & 0 \\ 0 & 0 & 1 \end{bmatrix}$$

Suppose that, across different cells, θ is distributed with mean $\mu_\theta = [\mu_{\theta,1} .. \mu_{\theta,3}]^T$ and covariance matrix $\Sigma_\theta = (s_{r,c})_{r,c=1..3}$. Regardless of the distribution, by the linearity of the model one has that

$$\mu_y = L\mu_\theta, \ \ \Sigma_y = L\, \Sigma_\theta L^T$$

For the sake of simplicity, we assume that the output statistics $\mu_y = [\mu_{y,1}\, \mu_{y,2}]^T$ and $\Sigma_y = (\sigma_{r,c})_{r,c=1,2}$ are known (while in practice, in the ME model inference framework, they would be estimated from population data).

The statistics of non-identifiable parameters are constrained by output statistics

In this section, we explore to what extent the different statistics of θ, μ_θ and Σ_θ, are constrained by the output statistics μ_y and Σ_y.

First-order moments. Because $\mu_y = L\mu_\theta$, it holds that $\mu_{y,1} = \mu_{\theta,1} + \mu_{\theta,2}$ and $\mu_{y,2} = \mu_{\theta,3}$. Thus, knowledge of μ_y allows reconstruction of the mean of θ_3, but the means of θ_1 and θ_2 are indistinguishable. Unless one is fixed, the other cannot be reconstructed.

Second-order moments. Because $\Sigma_y = L\, \Sigma_\theta L^T$, it must hold that

$$\sigma_{1,1} = s_{1,1} + 2s_{1,2} + s_{2,2} \tag{3.1}$$
$$\sigma_{1,2} = s_{1,3} + s_{2,3} \tag{3.2}$$
$$\sigma_{2,2} = s_{3,3} \tag{3.3}$$

This fixes $s_{3,3}$ equal to $\sigma_{2,2}$, but the other entries of Σ_θ are underdetermined. In addition, however, covariance matrices are positive semi-definite, i.e. all eigenvalues are (real and) nonnegative. By a known characterization of this class of matrices, this is equivalent to all principal minors (the determinants of all square matrices obtained by extracting the same rows and columns from the given matrix) being nonnegative. For our case study, among other inequalities, this criterion yields $s_{1,1} \geq 0$, $s_{2,2} \geq 0$ (variances are nonnegative) and $s_{1,1} * s_{2,2} - s_{1,2}^2 \geq 0$, which is equivalent to the two inequalities $s_{1,2} \leq \sqrt{s_{1,1}} * \sqrt{s_{2,2}}$ and $s_{1,2} \geq -\sqrt{s_{1,1}} * \sqrt{s_{2,2}}$. Used in conjunction with Eq. (3.1) this yields $\sigma_{1,1} \leq s_{1,1} + 2\sqrt{s_{1,1}} * \sqrt{s_{2,2}} + s_{2,2}$ and $\sigma_{1,1} \geq s_{1,1} - 2\sqrt{s_{1,1}} * \sqrt{s_{2,2}} + s_{2,2}$, or equivalently

$$\left(\sqrt{s_{1,1}} - \sqrt{s_{2,2}}\right)^2 \leq \sigma_{1,1} \leq \left(\sqrt{s_{1,1}} + \sqrt{s_{2,2}}\right)^2 \tag{4}$$

Thus, knowledge of $\sigma_{1,1}$ (output statistics) implies constraints on the variances of parameters θ_1 and θ_2. Similar constraints involving other entries of Σ_θ can be derived algebraically using (3.1)-(3.3) in conjunction with other implications of the positive-semidefiniteness of Σ_θ.

The resulting constraints can be graphically represented for particular values of Σ_y. Assuming for example that $\sigma_{1,1} = 1$, $\sigma_{1,2} = \sigma_{2,1} = -0.2$ and $\sigma_{2,2} = 0.5$, we obtain the plots in Fig. 1. For all pairs of entries of Σ_θ, scatter plots illustrate what values of these unknown entries are compatible with the known output statistics Σ_y for at least some values of the remaining entries of Σ_θ (i.e. satisfy $\Sigma_y = L\,\Sigma_\theta L^T$ with a positive-semidefinite Σ_θ). For example, Inequalities (4) determine the parabolic shape visible in the first-row, fourth-column plot. In Fig 2, a similar plot shows the relationships that must hold among $s_{1,1}$, $s_{2,2}$ and $s_{1,2}$, the second-order moments of the two unidentifiable parameters θ_1 and θ_2. This analysis shows that knowledge (or accurate estimate) of Σ_y, together with structural properties of covariance matrices, result in significant knowledge about the (yet underdetermined) values of the underlying statistics of interest, i.e. Σ_θ.

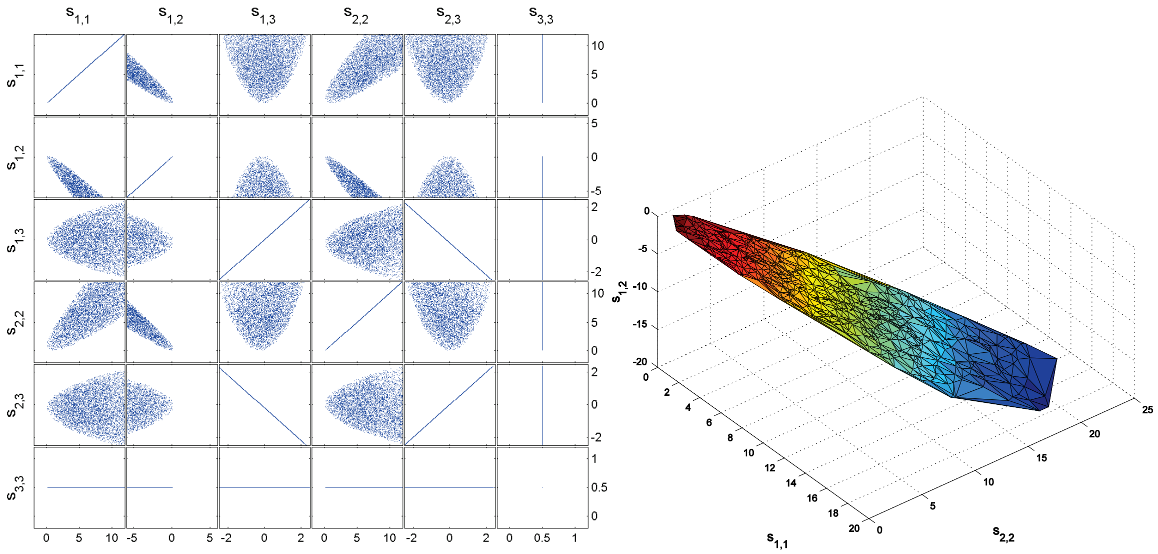

Figure 1. Second-order output statistics constraint second-order parameter statistics. (**Left**) Scatter plots of feasible value pairs for the unknown second-order statistics of parameter vector θ. For the given $\boldsymbol{\Sigma_y}$, all possible $\boldsymbol{\Sigma_\theta}$ are computed by first determining the affine space of symmetric solutions of the linear equation $\boldsymbol{\Sigma_y = L\Sigma_\theta L^T}$. Then, $\mathbf{10^6}$ candidate $\boldsymbol{\Sigma_\theta}$ are generated at random from within this space, and only the positive semidefinite solutions (*i.e.* the solutions with nonnegative eigenvalues) are retained and reported in the plot. (**Right**) Surface of feasible value triples for the unknown (joint) second-order statistics of the unknown parameters $\boldsymbol{\theta_1}$ and $\boldsymbol{\theta_2}$. Sample solution triplets are obtained by the method described above, and the plotted solution surface is obtained from the samples by triangulation.

The statistics of non-identifiable parameters are constrained by correlations between identifiable and non-identifiable parameters

We now pose the question how correlation between an identifiable parameter and a non-identifiable one may help the estimation of the latter. For simplicity let $\mu_\theta = 0$ (arguments below can be generalized to $\mu_\theta \neq 0$). Consider again the case where the identifiable parameter θ_3 is perfectly determined via y_2 by the observation of the single-cell output y. Regardless of the additional information provided by y_1, what can we say about, *e.g.*, θ_1? From the theory of linear estimation, the optimal linear estimator of θ_1, which is also optimal over all possible estimators in the Gaussian case, is $\theta_1^* = s_{1,3}s_{3,3}^{-1}\theta_3$, and the variance of the estimation error is

$$var(\theta_1^* - \theta_1) = s_{1,1} - s_{1,3}s_{3,3}^{-1}s_{3,1} = s_{1,1} - s_{1,3}^2/s_{3,3}$$

Thus, relative to the a priori variance $s_{1,1}$, observation of $y_2 = \theta_3$ decreases the uncertainty about $\theta_1$by the amount $s_{1,3}^2/s_{3,3}$, which is positive if the correlation $s_{1,3}$ between θ_1 and θ_3 is nonzero. The residual uncertainty about θ_1 is captured by the so-called Fraction of Unexplained Variance, defined as

$$FUV = 1 - {s_{1,3}^2}/{s_{1,1}s_{3,3}}$$

The larger the correlation between θ_1 and θ_3, the smaller the residual uncertainty about θ_1 given the knowledge of θ_3. Because $s_{1,1}$, $s_{3,3}$ and $s_{1,3}$ are only partially determined by Σ_y, the FUV cannot be computed uniquely. For the case of the previous section, however, we computed the average FUV over all sampled solutions Σ_θ compatible with Σ_y. We found that

$$average\ FUV \cong 0.75$$

and we found this number rather insensitive to the width of the sample space. That is, in absence of detailed information about Σ_θ, for the given Σ_y the sole knowledge of θ_3 is expected to contribute to 25% of the knowledge of θ_1. This analysis shows that indeed joint distributions may help refine the knowledge of non-identifiable parameters given the observation of identifiable parameters correlated with them.
