# Supplementary figures and images for: What Population Reveals about Individual Cell Identity: Single-Cell Parameter Estimation of Models of Gene Expression in Yeast
Source: PLoS Comput Biol. 2016 Feb 9;12(2):e1004706. doi: 10.1371/journal.pcbi.1004706 (PMC4747589; doi:10.1371/journal.pcbi.1004706)

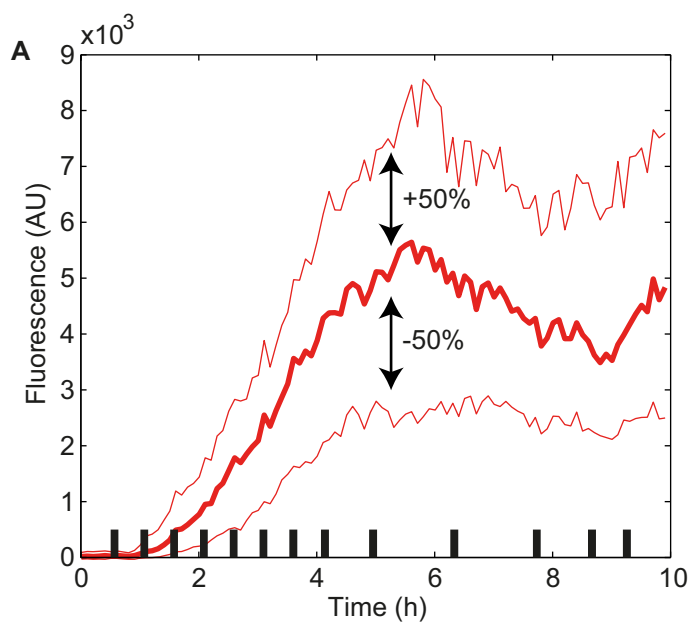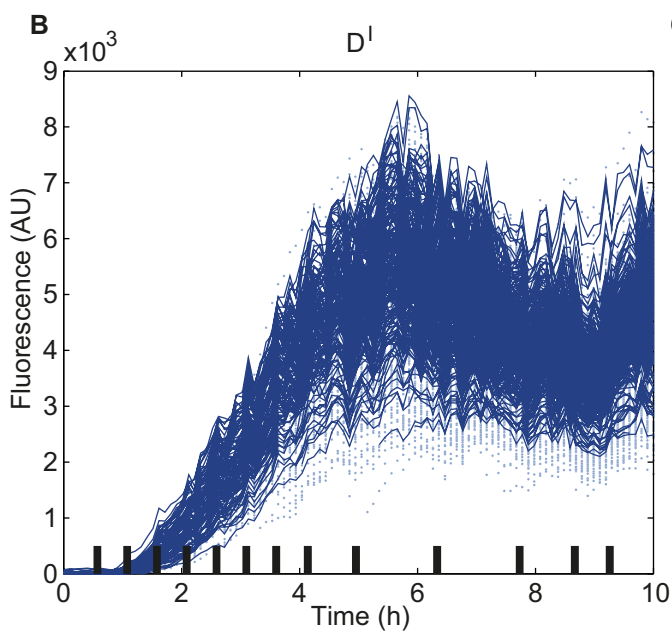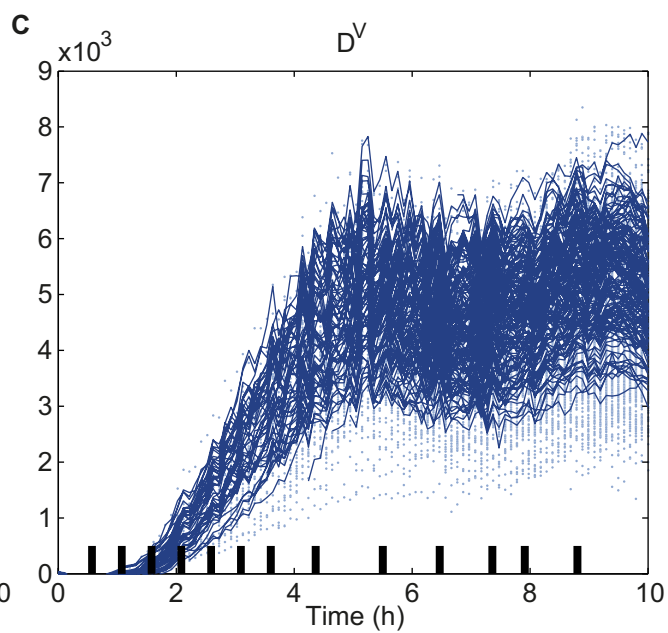

Supplement: S1 Fig — A. Minimum, maximum and average cellular fluorescence levels in the identification dataset DI. Back bars represent input shocks. B. Set of single cell trajectories present in the identification dataset DI (solid lines). Samples that did not pass all quality tests described in S1 Text appear as light blue dots. C. Set of single cell trajectories present in the validation dataset DV. (PDF) [file pcbi.1004706.s006.pdf]

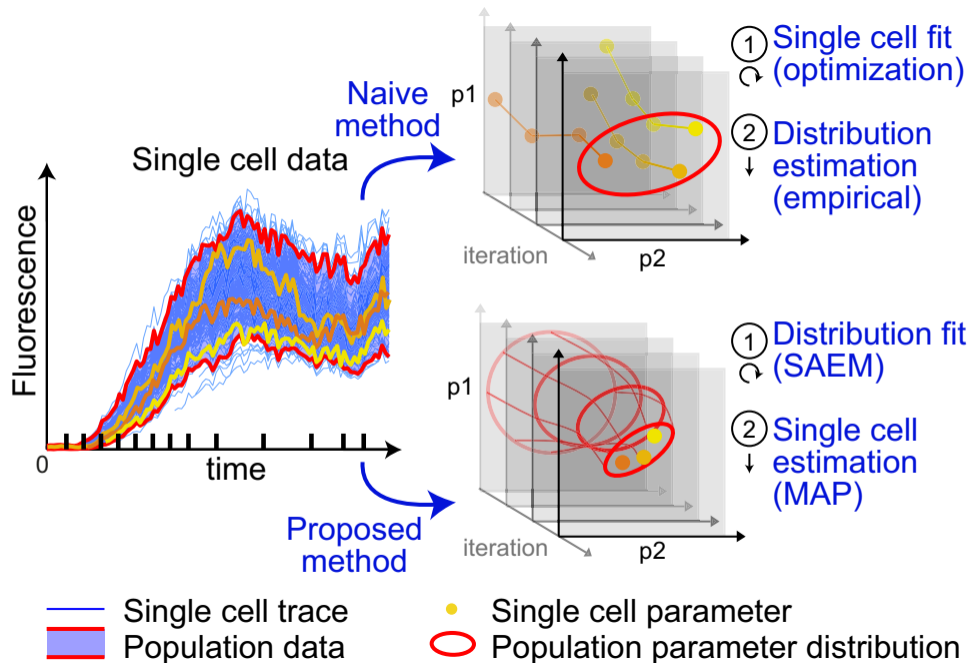

Supplement: S2 Fig — In the naive approach, optimization is used to seek -for each cell- parameter values fitting the individual behavior of the cell via residual minimization (top, step 1). The distribution describing all of the estimated parameter values is then deduced (top, step 2). In the proposed method, the SAEM tool is used to infer a distribution that explains the set of individual behaviors at the distribution level (bottom, step 1). Parameter values for single cells are then estimated based on the particular behavior of the cell and the inferred distribution for the population, using maximum a posteriori estimation (bottom, step 2). (PDF) [file pcbi.1004706.s007.pdf]

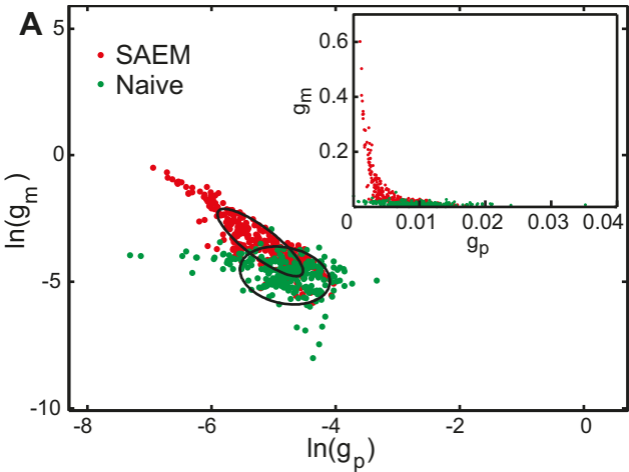

**B**

|       | Parameter distribution structure | Parameter distribution spread |
|-------|----------------------------------|-------------------------------|
| SAEM  | 0.62                             | 6.7                           |
| Naive | 0.19                             | 37                            |

Supplement: S3 Fig — A. 2D plot describing the distribution of the (logarithm of) single-cell parameters for two parameters (insert: same data shown in natural scale). The ellipses represent the region in which 50% of the parameters are distributed. B. Two metrics were computed to quantify the difference in the structure of the parameter distributions at a more global level. The first metric was the average of the coefficients of the variation matrix (i.e. of the off-diagonal terms covij/(μiμj) between the parameters of the model; this represents the amount of structure in the parameter distribution and shows that SAEM yielded a more structured parameter distribution. The second metric was the volume in the parameter space of the 95%-confidence ellipsoid associated with the covariance matrix. This yields a measure of the typical volume of parameter space occupied by the parameter distribution, and therefore, quantifies the spread of the parameter distributions. This showed that the SAEM approach described the population with a smaller distribution. (PDF) [file pcbi.1004706.s008.pdf]

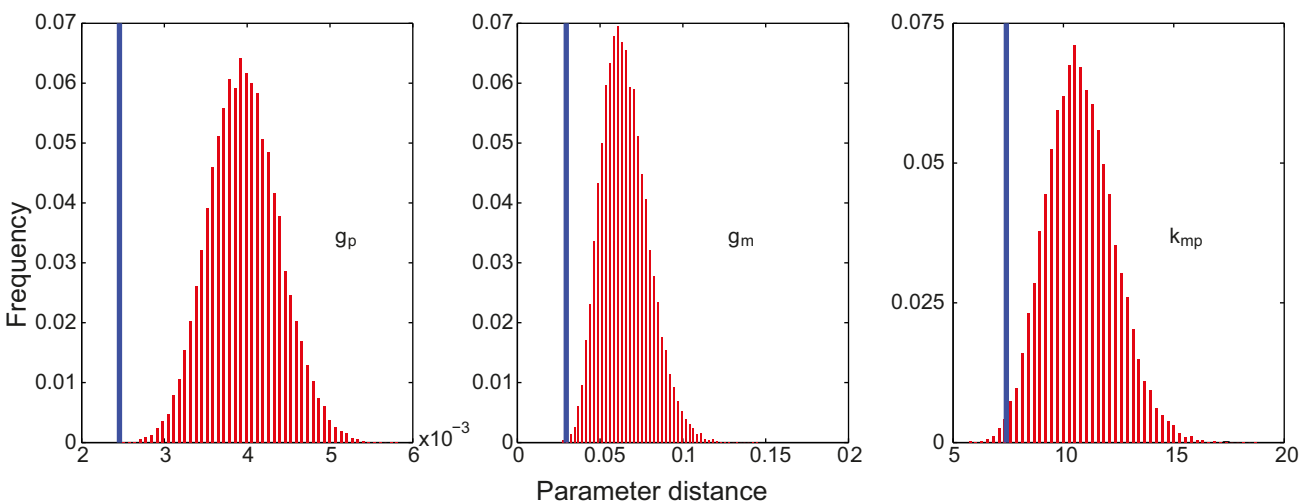

Supplement: S4 Fig — The blue bar represent the average distance in parameters between 55 mother-daughter pairs from experiment DI. The red distribution is obtained by bootstrapping 20000 sets of 55 random pairs of cells (from the same experiment). We see that the distance is very significantly smaller for mother-daughter pairs. (PDF) [file pcbi.1004706.s009.pdf]
