# Supplementary material for: What Population Reveals about Individual Cell Identity: Single-Cell Parameter Estimation of Models of Gene Expression in Yeast
Source: PLoS Comput Biol. 2016 Feb 9;12(2):e1004706. doi: 10.1371/journal.pcbi.1004706 (PMC4747589; doi:10.1371/journal.pcbi.1004706)
Supplement: S1 Table — (A) Initial values for the search have been obtained by global optimization (CMAES) on the mean behavior starting from literature-based parameters. The value of the delay τ has been fixed for all cells to its mean-cell. Therefore, statistics on its variability have been shaded. The dataset used is the identification set DI (B and C) The parameter search is initialized with parameter means extracted from the literature and a diagonal covariance matrix. The parameter search has been adapted to account for the structural non-identifiability relation of km and kp (only their product is relevant in single-cell models): the mean of kp is kept at a constant value during the search. No constraints are placed on its variance though. The value of the delay τ is estimated but is set identical for all cells. The dataset used for identification is DI (B) and DV (C). The relative standard errors of the estimated moments are typically less than 2%, with the exception of the estimate of SD[km] where it was 8%. (PDF) [file pcbi.1004706.s010.pdf]

A

Parameter	Inferred Values		Initials Values	Units	Parameter	Inferred Values		Initials Values
	Nat. Values	Log. Values	Log. Values			Nat. Values	Log. Values	Log. Values
$E[k_m]$	5.47	1.61	1.49	min^{-1}	$Corr(k_m g_m)$	0.128	0.167	NA
$E[g_m]$	$1.38 \cdot 10^{-2}$	-4.75	-4.74	min^{-1}	$Corr(k_m k_p)$	-0.172	-0.196	NA
$E[k_p]$	1.23	$7.81 \cdot 10^{-2}$	$5.66 \cdot 10^{-2}$	min^{-1}	$Corr(k_m g_p)$	0.297	0.328	NA
$E[g_p]$	$9.76 \cdot 10^{-3}$	-4.82	-4.81	min^{-1}	$Corr(k_m \tau)$	0	0	NA
$E[\tau]$	23.4	3.15	3.15	min	$Corr(g_m k_p)$	0.342	0.424	NA
$SD[k_m]$	2.41	0.421	NA	min^{-1}	$Corr(g_m g_p)$	-0.136	-0.205	NA
$SD[g_m]$	$1.73 \cdot 10^{-2}$	0.971	NA	min^{-1}	$Corr(g_m \tau)$	0	0	NA
$SD[k_p]$	0.667	0.508	NA	min^{-1}	$Corr(k_p g_p)$	0.216	0.244	NA
$SD[g_p]$	$6.60 \cdot 10^{-3}$	0.614	NA	min^{-1}	$Corr(k_p \tau)$	0	0	NA
$SD[\tau]$	0	0	NA	min	$Corr(g_p \tau)$	0	0	NA
ε_a	45.3	3.81	3.62	AU				
ε_b	$9.13 \cdot 10^{-2}$	-2.39	-1.51	-				

B

Parameter	Inferred Values		Initials Values	Units	Parameter	Inferred Values		Initials Values
	Nat. Values	Log. Values	Log. Values			Nat. Values	Log. Values	Log. Values
$E[k_m]$	14.7	2.63	2.30	min^{-1}	$Corr(k_m g_m)$	0.320	0.432	0
$E[g_m]$	$6.00 \cdot 10^{-2}$	-3.45	-1.22	min^{-1}	$Corr(k_m k_p)$	-0.0647	-0.0753	0
$E[k_p]$	1.19	$-5.45 \cdot 10^{-2}$	$-5.45 \cdot 10^{-2}$	min^{-1}	$Corr(k_m g_p)$	-0.324	-0.376	0
$E[g_p]$	$6.45 \cdot 10^{-3}$	-5.22	-5.52	min^{-1}	$Corr(k_m \tau)$	0	0	0
$E[\tau]$	37.0	3.61	3.40	min	$Corr(g_m k_p)$	0.627	0.746	0
$SD[k_m]$	4.80	0.319	0.330	min^{-1}	$Corr(g_m g_p)$	-0.416	-0.843	0
$SD[g_m]$	$9.66 \cdot 10^{-2}$	1.13	0.330	min^{-1}	$Corr(g_m \tau)$	0	0	0
$SD[k_p]$	0.902	0.674	0.330	min^{-1}	$Corr(k_p g_p)$	-0.289	-0.382	0
$SD[g_p]$	$4.12 \cdot 10^{-3}$	0.585	0.330	min^{-1}	$Corr(k_p \tau)$	0	0	0
$SD[\tau]$	0	0	0	min	$Corr(g_p \tau)$	0	0	0
ε_a	63.9	4.16	5.99	AU				
ε_b	$8.67 \cdot 10^{-2}$	-2.45	-1.20	-				

C

Parameter	Inferred Values		Initials Values	Units	Parameter	Inferred Values		Initials Values
	Nat. Values	Log. Values	Log. Values			Nat. Values	Log. Values	Log. Values
$E[k_m]$	9.33	2.10	2.30	min^{-1}	$Corr(k_m g_m)$	0.604	0.733	0
$E[g_m]$	$4.17 \cdot 10^{-2}$	-3.80	-1.22	min^{-1}	$Corr(k_m k_p)$	0.329	0.359	0
$E[k_p]$	1.08	$-5.45 \cdot 10^{-2}$	$-5.45 \cdot 10^{-2}$	min^{-1}	$Corr(k_m g_p)$	$2.52 \cdot 10^{-2}$	$2.99 \cdot 10^{-2}$	0
$E[g_p]$	$4.36 \cdot 10^{-3}$	-5.65	-5.52	min^{-1}	$Corr(k_m \tau)$	0	0	0
$E[\tau]$	50.4	3.92	3.40	min	$Corr(g_m k_p)$	0.578	0.706	0
$SD[k_m]$	5.08	0.509	0.330	min^{-1}	$Corr(g_m g_p)$	-0.247	-0.458	0
$SD[g_m]$	$6.57 \cdot 10^{-2}$	1.12	0.330	min^{-1}	$Corr(g_m \tau)$	0	0	0
$SD[k_p]$	0.605	0.521	0.330	min^{-1}	$Corr(k_p g_p)$	$-4.05 \cdot 10^{-2}$	$-4.88 \cdot 10^{-2}$	0
$SD[g_p]$	$3.17 \cdot 10^{-2}$	0.652	0.330	min^{-1}	$Corr(k_p \tau)$	0	0	0
$SD[\tau]$	0	0	0	min	$Corr(g_p \tau)$	0	0	0
ε_a	68.6	4.23	5.99	AU				
ε_b	$6.42 \cdot 10^{-2}$	-2.75	-1.20	-				
